# Proteomic and metabolomic analysis of the cellular biomarkers related to inhibitors tolerance in *Zymomonas mobilis* ZM4

**DOI:** 10.1186/s13068-018-1287-5

**Published:** 2018-10-16

**Authors:** Dongdong Chang, Zhisheng Yu, Zia Ul Islam, W. Todd French, Yiming Zhang, Hongxun Zhang

**Affiliations:** 1 College of Resources and Environment, University of Chinese Academy of Sciences, 19 A Yuquan Road, Shijingshan District, Beijing, 100049 People’s Republic of China; 2 Department of Sustainable Bioproducts, Mississippi State University, Mississippi State, MS 39762 USA; 3 Dave C. Swalm School of Chemical Engineering, Mississippi State University, P.O. Box 9595, Mississippi State, MS 39762 USA; 4 Environmental Protection Bureau, Shunyi District, Beijing, 101300 People’s Republic of China

**Keywords:** Proteomics, Metabolomics, *Z. mobilis*, Biomass, Inhibitors, Bioethanol

## Abstract

**Background:**

Toxic compounds present in both the hydrolysate and pyrolysate of lignocellulosic biomass severely hinder the further conversion of lignocellulose-derived fermentable sugars into useful chemicals by common biocatalysts like *Zymomonas mobilis*, which has remarkable advantages over yeast. Although the extra detoxification treatment prior to fermentation process can help biocatalysts to eliminate the inhibitory environment, it is not environment friendly and cost effective for industrial application. As also reported by previous studies, an ideal and holistic approach to solve this issue is to develop microbial strains with inhibitor tolerance. However, previously engineered strains had the limitation that they could not cope well with the synergistic effect of multiple inhibitors as they are resistant only to a single inhibitor. Hence, understanding the universal cellular responses of *Z. mobilis* to various inhibitors may guide the designing of rational strategies to obtain more robust engineered strains for biofuel production from lignocellulosic biomass.

**Results:**

Quantitative proteomics and metabolomics approaches were used to determine the cellular responses of *Z. mobilis* ZM4 to representative biomass-derived inhibitors like formic acid, acetic acid, furfural, 5-hydroxymethylfurfural, and phenol. The differentially expressed proteins identified under the challenge of single and combined inhibitors were involved in cell wall/membrane biogenesis, energy production, DNA replication, DNA recombination, DNA repair, DNA transcription, RNA translation, posttranslational modification, biosynthesis of amino acids, central carbon metabolism, etc. Metabolomics analysis showed that the up- or down-regulation pattern of metabolites was changed consistently with that of relevant proteins.

**Conclusion:**

Fifteen up-regulated proteins (e.g., Isopropylmalate isomerase LeuC, transcription-repair-coupling factor Mfd, and phosphoglucose isomerase PGI) and thirteen down-regulated proteins (e.g., TonB-dependent transporter ZMO1522, transcription termination factor Rho, and S1/P1 nuclease ZMO0127) were identified as candidate proteins related to all the stress conditions, implying that these proteins are potential biomarkers for the improvement of *Z. mobilis* ZM4 to resist complex biomass-derived inhibitors. These data can be used to generate a database of inhibitor-tolerance biomarkers, which could provide a basis for engineering *Z. mobilis* that would be able to grow in the presence of multiple inhibitors and directly ferment the biomass-derived sugars into biofuels.

**Electronic supplementary material:**

The online version of this article (10.1186/s13068-018-1287-5) contains supplementary material, which is available to authorized users.

## Background

The quest for sustainable and environmentally friendly sources of energy for industrial and individual utilization has attracted a significant amount of attention in recent years. To reduce dependence on continuously depleting fossil fuel reserves and address the issue of greenhouse gases emissions, lignocellulosic biomass is the most abundant biopolymer on the planet from which renewable fuels can be produced without interfering with the human food chain. After its pretreatment by physical, chemical, or biochemical processes, biomass-derived sugars can be transformed into biofuels via thermochemical or biochemical pathways [1]. However, apart from the fermentable sugars, many undesirable toxic compounds that are inhibitory to the microbial biocatalysts are also produced during biomass pretreatment and saccharification processes. Inhibitors such as low-molecular weight organic acids, furans, and aromatic compounds are produced during the hydrolysis or pyrolysis treatment of the biomass feedstock. These inhibitors have adverse effects on the ethanologenic biocatalysts like *Zymomonas mobilis*, *Saccharomyces cerevisiae*, and engineered *Escherichia coli* [2–5]. Inhibitor removal processes are applied to overcome this problem of microbial inhibition and increase the yield of biofuels from the hydrolysate or pyrolysate media [3, 6–9], but they add to the overall cost of the process. To improve the overall economics of producing biofuels from biomass hydrolysates or pyrolysates, a biocatalyst with inhibitor-resistant pathways would negate the need for expensive inhibitor-removal processes.

Among the various biocatalysts, *Z. mobilis* is regarded as the desired platform for future biorefineries due to its many desirable industrial characteristics such as Entner–Doudoroff (ED) pathway for glycolysis, low cell mass formation, high substrate conversion efficiency, high specific cell surface area, high specific productivity and yield, wide pH range, and high ethanol tolerance [5, 10, 11]. Unfortunately, it is more susceptible to the inhibitors than other biocatalysts like ethanologenic *E. coli* and *S. cerevisiae* [3, 6, 9, 12, 13]. Some previous studies have evaluated the effects of inhibitory compounds on the growth and fermentation ability of *Z. mobilis* ATCC 10988 [12], *Z. mobilis* ZM4 [14, 15], *Z. mobilis* CP4/pZB5 [16], and *Z. mobilis* 8b [17]; these studies on the toxicity of inhibitors provided a basis for further studies on the strain improvement of *Z. mobilis*.

Recently, genetic approaches, including forward and reverse genetics, have been applied to develop the inhibitor-resistant *Z. mobilis* strains [11, 14, 18–22]. Using reverse genetics approaches, researchers were successful in developing a few acetate-resistant [18–20] and phenols-resistant strains [22]. The overexpression of sodium-proton antiporter gene *nhaA* (ZMO0119) and RNA chaperone gene *hfq* (ZMO0347) improved mutant strain ZM4 (AcR) ability to grow under a relatively high concentration of sodium acetate [18–20], and the overexpression of reductase genes ZMO1116, ZMO1696, and ZMO1885 also improved the resistance of ZM4 to phenolic aldehyde inhibitors [22]. Using forward genetics, strategies like adaptive evolution was also performed to get mutants that resisted inhibition by furfural, acetic acid, and multiple inhibitors in corn stover hydrolysate [14, 21], although these mutants required further genomic re-sequencing analysis for the identification of potential mutation sites. A review of previous researches has demonstrated that researchers have mainly focused on developing strains resistant to a single inhibitor. Although it is meaningful to develop strains resistant to a certain inhibitor in lab conditions, in practical industrial applications, different inhibitors are often present together in the hydrolysate or pyrolysate of biomass and necessitate the development of mutants resistant to all the potential inhibitors present in these media. Considering the synergistic effect of the inhibitors, robust engineered strains capable of resisting multiple inhibitors should be explored to get around the limitations exhibited by strains only resistant to a single inhibitor.

Omics technologies involved in reverse genetics approaches, such as transcriptomics, proteomics, and metabolomics will improve our understanding of biological processes and have become the new mantra in molecular research [23]. Thus, the application of these technologies could help us to discern how microorganisms respond to different environmental stresses and formulate ways to improve or modify their genotype to let them perform optimally in the presence of inhibitors. Previous transcriptomics and proteomics studies reported that, increased expression of genes *fucO*, *ucpA*, or *pntAB* and deletion of gene *yqhD* were able to confer furfural resistance to *E. coli* [24]; furfural resistance of different microorganisms also could be acquired by overexpression of aldo/keto reductase (AKR) and short-chain dehydrogenase/reductase (SDR) in *Clostridium beijerinckii* [25], overexpression of transcription activator Msn2 in *S. cerevisiae* [26], and overexpression of efflux-like permease in *Corynebacterium glutamicum* [27]. Additionally, overexpression of sigma factor RpoS, glutaminase YbaS, glutamate or arginine decarboxylases, and these decarboxylases attendant antiporters could help *E. coli* to improve acid resistance [28, 29]; overexpression of Sso2p protein and laccase could also confer phenolic compounds resistance to *S. cerevisiae* [30]. Similarly, transcriptomic profiles of *Z. mobilis* have also been established under certain inhibitory conditions [5, 22, 31–34] that provide an understanding of the response of *Z. mobilis* to that inhibitor. However, the specific molecular mechanisms or biomarker molecules of *Z. mobilis* in response to various lignocellulosic biomass-derived inhibitors like furans, organic acids and phenols are still not well understood, and this is an obstacle for developing inhibitors-resistant strains of *Z. mobilis* through genetic engineering.

In this study, *Z. mobilis* ZM4 was challenged with individual or combinations of five representative biomass-derived inhibitory compounds: two furans, furfural and 5-hydroxymethylfurfural (5-HMF); two organic acids, acetic acid, and formic acid; one aromatic compound, phenol. Subsequently, the cellular responses of *Z. mobilis* ZM4 to individual and combined inhibitors at proteomics and metabolomics levels were determined to elucidate the biomarkers related to inhibitor-resistant functions of *Z. mobilis* ZM4. The present data will help in the understanding of the molecular mechanism for cellular tolerance to biomass-derived inhibitors and provide insight towards strain improvement by genetic engineering or synthetic biology.

## Results and discussion

### Effects of single or combined inhibitors on cell growth of *Z. mobilis* ZM4

**Fig. 1:**
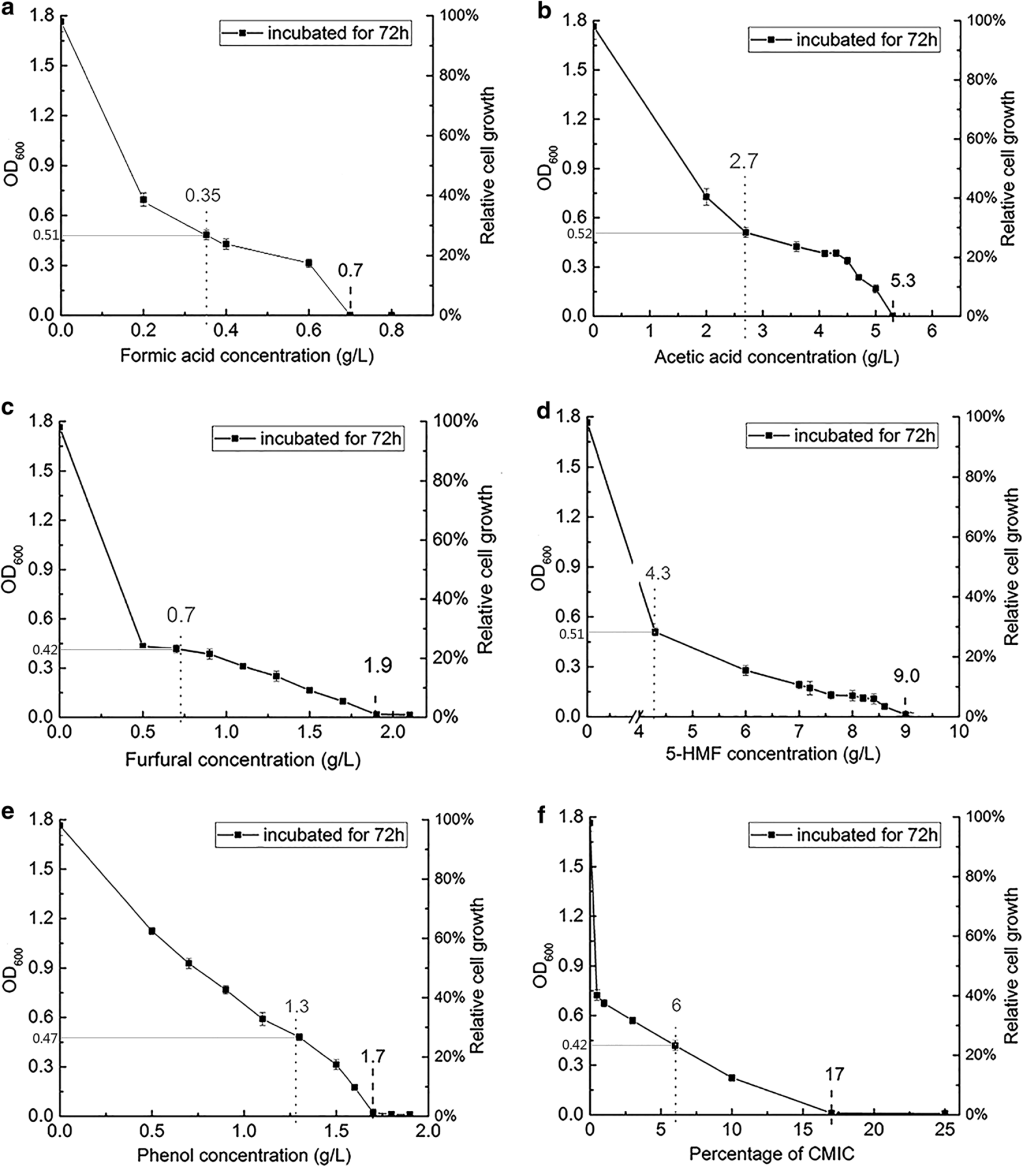
Inhibitory profiles of individual and combined inhibitors on *Zymomonas mobilis* ZM4. **a** Cells challenged by formic acid. **b** Cells challenged by acetic acid. **c** Cells challenged by furfural. **d** Cells challenged by 5-HMF. **e** Cells challenged by phenol. **f** Cells challenged by combined inhibitors; Percentage of CMIC, it is the percentage that all of the individual MIC (minimum inhibitory concentration) of each inhibitor were multiplied by, then combined into a single culture; 100% CMIC denotes the combination of 1X MIC of each inhibitor. Dark dotted line denotes the concentration of certain inhibitor completely blocked cell growth. Gray dotted line denotes the concentration of certain inhibitor was chosen for further proteomics and metabolomics analysis

The impact of different concentrations of inhibitors on cell growth of *Z. mobilis* was determined by measuring changes to OD_600_ value when compared to the control. OD_600_ value of *Z. mobilis* ZM4 grown in the absence of these inhibitors reached a maximum of 1.77 at 34 h post-inoculation, which was designed as the maximum final cell density of the control. Within 72-h incubation time, the final cell densities obtained with formic acid concentrations < 0.20 g/L, acetic acid concentrations < 2.00 g/L, furfural concentrations < 0.30 g/L, 5-HMF concentrations < 2.50 g/L, and phenol concentrations < 0.50 g/L were the same as the control at 34 h. However, it is apparent that the inhibitors with increasing concentrations negatively affect the cell growth rate (Additional file 1: Fig. S1). At formic acid concentrations between 0.20 and 0.70 g/L, acetic acid concentrations between 2.00 and 5.30 g/L, furfural concentrations between 0.30 and 1.90 g/L, 5-HMF concentrations between 2.50 and 9.00 g/L, and phenol concentrations between 0.50 and 1.70 g/L, the final cell densities decreased with the increasing concentrations of inhibitors; the final cell densities under different conditions peaked mostly within 72-h incubation, although in some cases taking more than 72 h (Additional file 1: Fig. S1). The final cell densities measured at 72-h time point at different concentrations of these inhibitors were plotted against inhibitor concentrations in Fig. 1, to allow for better visualization of the data, and accordingly, the relative final cell density was calculated as a percentage of OD_600_ value divided by the value obtained from the control.

Eventually, formic acid (Fig. 1a), acetic acid (Fig. 1b), furfural (Fig. 1c), 5-HMF (Fig. 1d), and phenol (Fig. 1e) completely blocked the cell growth of ZM4 at the minimum inhibitory concentration (MIC) levels (0.70 g/L, 5.30 g/L, 1.90 g/L, 9.00 g/L, and 1.70 g/L, respectively) as determined by no change in OD measurements. It was previously reported that the cell growth of *Z. mobilis* 8b was completely blocked in the presence of 25.00 g/L acetic acid, 5.00 g/L furfural, and 8.00 g/L 5-HMF, respectively [15]; growth of *Z. mobilis* ATCC29191 was completely inhibited in the presence of 11.00 g/L acetic acid [35]. Herein, different MICs of acetic acid, furfural, and 5-HMF were exhibited, implying that different strains have different physiological responses to the inhibitors. Therefore, it is necessary for us to determine the MIC of each inhibitor. Inhibitors present in the biomass-derived liquid usually act synergistically [15, 17], and this phenomenon was also confirmed by this study’s results in that concentration higher than 17% of the combination of the MIC of each inhibitor (CMIC) completely blocked the cell growth (Fig. 1f).

To conduct the proteomics and metabolomics analysis, the concentrations of inhibitors were chosen in such a way to inhibit the cell growth to an appropriate level but not blocking it completely; that is, 0.35 g/L formic acid, 2.70 g/L acetic acid, 0.70 g/L furfural, 4.30 g/L 5-HMF, 1.30 g/L phenol, and 6% of the CMIC of each inhibitor (a combination of 0.04 g/L formic acid, 0.32 g/L acetic acid, 0.11 g/L furfural, 0.54 g/L 5-HMF, and 0.10 g/L phenol). These inhibitors concentrations were chosen because they all resulted in a cell density with a final OD_600_ value of 0.47 ± 0.05 after 72-h incubation (Fig. 1). Fermentation data are presented in Additional file 1: Fig. S2 for *Z. mobilis* ZM4 grown in the presence of the inhibitors with the concentrations mentioned above. As shown in Additional file 1: Fig. S2, these inhibitors repressed the cell growth, glucose consumption, and ethanol yield apparently. Glucose substrates were not completely consumed under all the stress conditions, leading to lower ethanol yields than the control (Additional file 1: Fig. S2). It was previously reported that the cell growth rate, final cell density, glucose consumption, and ethanol production of *Z. mobilis* 8b were also negatively affected by various inhibitors [17]. However, glucose substrates were all consumed by *Z. mobilis* 8b in the presence of acetic acid, furfural, and 5-HMF, which are different from our results. One explanation might be the concentrations of the inhibitors used were different; another one might be the inocula of *Z. mobilis* 8b they used were concentrated ones with high cell density, and therefore, could enhance the cellular capacity to bind extracellular inhibitors and reduce the bioavailability of inhibitors [17].

To minimize interference by changes in the cellular proteins or metabolites composition induced by the (poorly defined) factors that limit exponential growth of these cells in the transition to stationary phase [36], cells challenged with different inhibitors should be harvested at the early- or mid-logarithmic growth phase for proteomics and metabolomics analysis. Here, *Z. mobilis* cell pellets, under the stress of these inhibitors, were harvested at early-logarithmic growth phase with an OD_600_ value of 0.17 ± 0.02 (Additional file 1: Fig. S2A). To reach this cell density, *Z. mobilis* took ~ 5 h to grow in the control culture, ~ 24 h in the formic acid treated culture, ~ 33 h in the acetic acid treated culture, ~ 18 h in the furfural treated culture, ~ 21 h in the 5-HMF treated culture, ~ 23 h in the phenol-treated culture, and ~ 25 h in the combined inhibitors treated culture, respectively.

### Overview of quantitative proteomics and metabolomics

Quantitative proteomics was used to compare the proteomic differences of *Z. mobilis* ZM4 cellular proteins in the presence and absence of inhibitors. The proteomic analysis identified 142,987 spectra, which were matched to 1366 unique proteins (Additional file 2: Sheet S1). With a cutoff of more than 1.5-fold change and a *p* value of statistical significance less than 0.05, numbers of both up-regulated and down-regulated differentially expressed proteins (DEPs) in all the groups were determined and shown in Additional file 1: Fig. S3, with group C showing the most abundant DEPs with 258 up-regulated and 195 down-regulated proteins, followed by group F with 245 up-regulated and 179 down-regulated proteins.

**Fig. 2:**
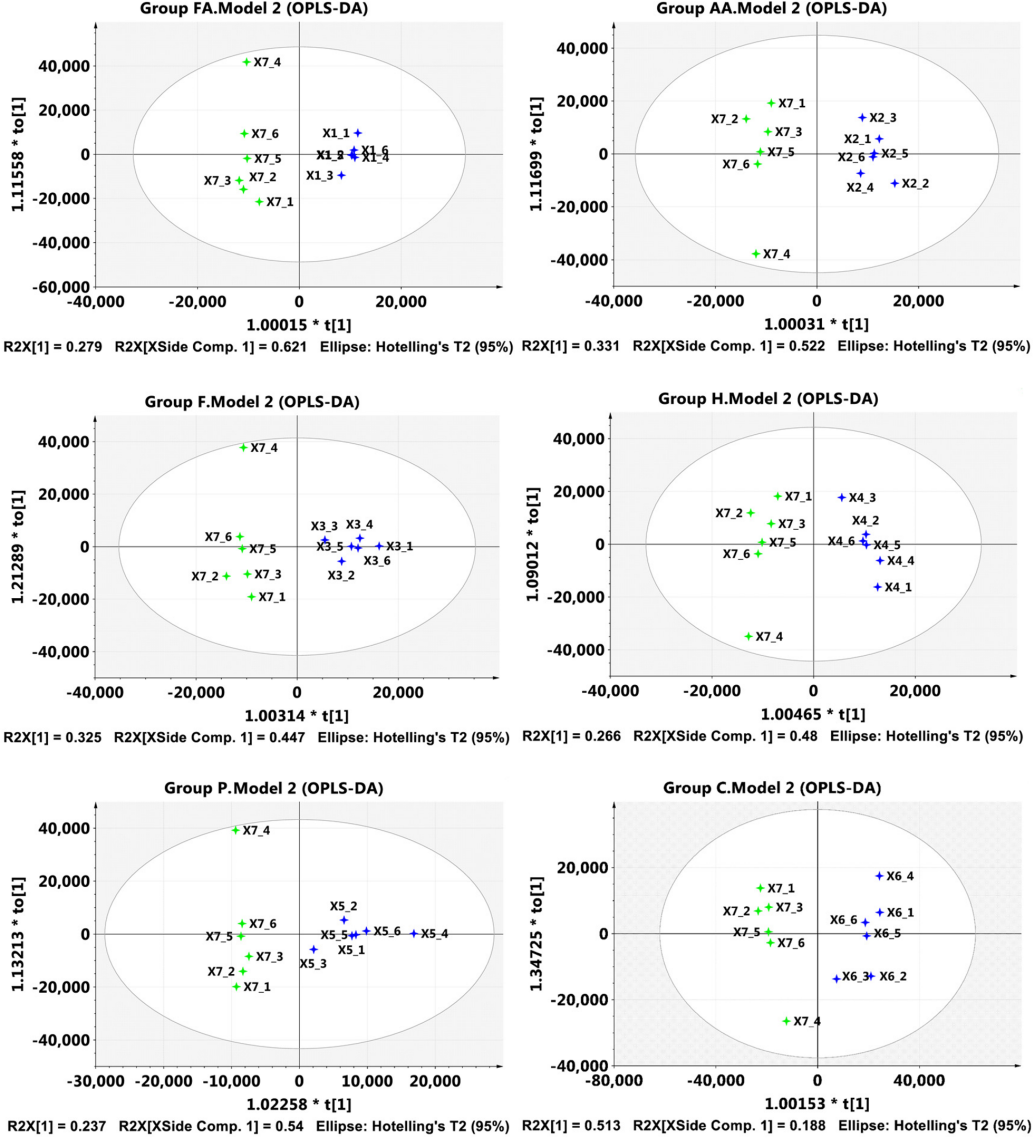
OPLS-DA analysis of the total metabolites identified in the inhibitor-treated groups and control. Group FA, cells treated by formic acid. Group AA, cells treated by acetic acid. Group F, cells treated by furfural. Group H, cells treated by 5-HMF. Group P, cells treated by phenol. Group C, cells treated by combined inhibitors. X1_1 to X1_6 were six duplications of metabolites obtained in formic acid-treated group, X2_1 to X2_6 were those in acetic acid treated group, X3_1 to X3_6 were those in furfural treated group, X4_1 to X4_6 were those in 5-HMF treated group, X5_1 to X5_6 were those in phenol-treated group, X6_1 to X6_6 were those in combined inhibitors treated group, and X7_1 to X7_6 were those in control group. The horizontal direction (t [1]) indicates the separation of the two independent groups; the farther the distance on the horizontal axis, the greater the discrepancies of the differentially expressed metabolites of the two groups. The vertical direction (to [1]) indicates the separation within the reduplicates of a certain group, which is less important than the horizontal direction

The GC–MS analysis further elucidated the physiological status of *Z. mobilis* ZM4 indicated by metabolomic profiles. Seven sets of the metabolomics profiles each detected 168 metabolites for groups FA, AA, F, H, P, C, and CK, respectively (Additional file 3: Sheet S2). The metabolites obtained in the inhibitor-treated groups were separated from the control, and the separation was illustrated by the OPLS-DA score plot (Fig. 2); considering OPLS-DA analysis was better for data separation than PCA analysis (Additional file 1: Fig. S4). As also shown in Fig. 2, when two components were calculated, the cumulative R2X values were 0.900, 0.853, 0.772, 0.746, 0.777, and 0.701, respectively; R2Y values were 0.988, 0.971, 0.947, 0.954, 0.881, and 0.942, respectively; Q2Y values were 0.918, 934, 0.914, 0.903, 0.735, and 0.795, respectively. Biomarker metabolites that showed significant differences in relative abundance and statistical significance (VIP > 1, *p* value < 0.05) are listed in Table 1, wherein the up-regulated and down-regulated metabolites are also marked out.

**Table 1:** Biomarker metabolites identified in each inhibitor-treated group

Metabolites	VIP values					
	FA	AA	F	H	P	C
Glucose	5.41^#^	5.70^#^	\	\	\	\
Oxoproline	5.31	3.50	5.10	4.57	2.40^#^	2.42^#^
Glycerol	4.66	4.13	3.21	\	2.20^#^	1.78^#^
Alanine	3.88	\	\	\	\	3.91^#^
Gluconic lactone	2.77^#^	\	1.81	\	4.30	3.25^#^
d-Glyceric acid	2.52	2.30	1.64	1.60	2.34	\
Leucine	2.29	\	1.92^#^	\	\	3.11^#^
3-Hydroxypropionic acid	1.94	1.76	\	\	\	\
Lysine	1.67	\	1.01	\	\	\
2-hydroxypyridine	1.59	1.19	1.29	\	1.02	\
Phenylalanine	1.53	1.20	1.09	1.26	\	\
Inosine	1.37	1.61	1.85^#^	\	\	\
Trehalose	1.37	\	1.50^#^	\	1.02^#^	2.32^#^
Lactic acid	1.15	\		\	1.19	\
Ribose	1.13	1.32	1.21	1.16	\	\
Gluconic acid	1.11^#^	4.95^#^	3.86^#^	6.86^#^	6.01	6.70^#^
Tyrosine	1.05	\	\	\	\	\
l-Allothreonine	1.03	1.30^#^	1.57	\	\	3.25^#^
*O*-Phosphoserine	1.02^#^	\	\	\	\	\
Phosphate	\	2.58	3.36	2.81	\	\
Isoleucine	\	2.10^#^	1.99^#^	2.31^#^	2.34^#^	2.36^#^
Linoleic acid methyl ester	\	2.04^#^	3.21^#^	2.22^#^	2.14	1.33^#^
Glycine	\	1.69^#^	1.65^#^	1.75^#^	1.93^#^	1.87^#^
Stearic acid	\	1.53^#^	1.89^#^	\	\	1.33^#^
Valine	\	1.52^#^	4.10^#^	2.35^#^	2.35^#^	2.19^#^
Proline	\	1.49^#^	1.58^#^	1.50^#^	1.95^#^	1.15^#^
Oleic acid	\	1.43^#^	1.10^#^	\	\	1.29^#^
Serine	\	1.43^#^	1.04^#^	1.15^#^	2.30^#^	2.10^#^
Succinic acid	\	1.17^#^	1.84^#^	1.43^#^	1.25^#^	1.32^#^
Tyrosine	\	1.02	\	\	\	\
Dihydroxyacetone	\	\	1.52^#^	2.69^#^	5.72^#^	1.35^#^
Aspartic acid	\	\	1.29	1.16	1.50	\
Palmitic acid	\	\	1.00	\	\	\
1,2-Cyclohexanedione	\	\	\	4.56^#^	\	2.11^#^

*VIP* variable importance to projection, obtained by OPLS-DA analysis. *FA* cells treated by formic acid, *AA* cells treated by acetic acid, *F* cells treated by furfural, *H* cells treated by 5-HMF, *P* cells treated by phenol, *C* cells treated by combined inhibitors. Symbol “\” denotes the metabolite is not identified as a biomarker in the relevant group. Superscript symbol “#” denotes the metabolite is up-regulated. Metabolite without symbol denotes it is down-regulated

To get an insight of the gene ontology (GO) categories and KEGG pathway enrichment of these DEPs and metabolites, Additional file 1: Text S1 is presented in Additional file 1. The DEPs mainly participated in the metabolic process, constituted the cell part, and played roles in the catalytic activity, corresponding to the GO ontologies. Moreover, these DEPs and metabolites were enriched in metabolic pathway, biosynthesis of secondary metabolites pathway, and biosynthesis of antibiotics pathway, etc.

### Proteomic profiling of *Z. mobilis* ZM4 under different inhibitory stress

#### Cell wall/membrane/envelope biogenesis and cell motility-related proteins

Many proteins related to cell wall/membrane/envelope biogenesis and cell motility in the presence of inhibitors were differentially expressed when compared with the control. Efflux system of living cells that relies on efflux pumps, which are proteinaceous transporters localized in the cytoplasmic membrane, is an efficient system for the detoxification of external toxic compounds and internal damaging intermediates [22]. In the mentioned groups treated by different inhibitors (Additional file 4: Table S1), efflux proteins like ZMO0778, ZMO0779, and ZMO0780 were down-regulated; however, ZMO0282, ZMO0285, ZMO0964, ZMO0965, ZMO1430, and ZMO1529 were up-regulated. The expression of efflux protein ZMO0283 was down-regulated in response to weak acid stress, while up-regulated in response to furfural and phenolic compounds stress. These results like the up-regulation of ZMO0282, ZMO0283, ZMO0965, and ZMO1529 were in line with the transcriptomic results of *Z. mobilis* ZM4 treated by phenolic aldehydes [22], and so the down-regulation of ZMO0779 in line with the results treated by furfural [33]. The differentially expressed efflux proteins in different groups are quite diverse, implying that the roles of these proteins vary under the challenge of different inhibitors.

TonB-dependent transporters present in bacteria are required for active uptake of iron complexes, polypeptides, and carbohydrates [37]. Although iron assimilation is essential in some bacteria, *Z. mobilis* cells do not produce siderophores that are produced by most bacteria [38]. It was speculated that TonB-dependent transporter proteins could contribute to osmotic pressure resistance by transporting other unknown molecules [32]. Here, our results showed that TonB-dependent transporter proteins ZMO0128, ZMO0188, ZMO0979, ZMO1522, ZMO1694, and ZMO1717 were down-regulated (Additional file 4: Table S1). The down-regulation of ZMO0128 and ZMO1522 was in line with the results of challenging *Z. mobilis* 8b with acetate in the literature [39], wherein it was further speculated that down-regulation of the two TonB-dependent transporters might reduce energy needs for substrate uptake and reserve energy for stress responses.

Lipoproteins are important proteins functioning efficiently at the membrane–aqueous interface as structural proteins, membrane-bound enzymes, and transport proteins. These proteins like ZMO0166, ZMO1207, and ZMO1701 were up-regulated, while ZMO0908 and ZMO1439 were down-regulated. Bacterial ABC transporters which can transport various substrates across cellular membranes are essential in cell viability, virulence, and pathogenicity. The ABC transporter-related proteins were differentially expressed under different inhibitors, such as ZMO0183, ZMO1029, and ZMO1590 were up-regulated, while ZMO1017, ZMO1029, and ZMO1355 were down-regulated. Porin acting as membrane channel protein is large enough to allow passive diffusion. In our results, porin ZMO0847 was down-regulated in the presence of formic acid, phenol, and combined inhibitors.

Hopanoids, a class of pentacyclic triterpenoid lipids, are membrane components involved in regulating membrane fluidity and stability and maintaining membrane integrity and pH homeostasis [40]. In the current study, proteins involved in hopanoid biosynthesis and other terpenoid biosynthesis pathways like HpnJ, HpnH, Shc, Dxs1, IspH, IspA, Dxr, IspG, and IspB were down-regulated, while IspDF was up-regulated (Additional file 4: Table S1). Previously, it has been reported that proteins Dxs1, IspG, and IspA of *Z. mobilis* ZM4 were down-regulated in response to the stress of ethanol [34], and the same pattern was observed in response to the stress of various inhibitors in our study. However, HpnJ, HpnH, Shc, IspH, Dxr, and IspB were down-regulated, while IspDF was up-regulated in our results, which is in contrast to the pattern that was demonstrated under the stress of ethanol [31]. In fact, ethanol could facilitate the formation of hopanoids [41], and therefore, it is reasonable to expect that most of the triterpenoid biosynthesis-related proteins were up-regulated in response to ethanol [34]. Additionally, a transcriptomic study also demonstrated that furfural could result in a down-regulation of a series of triterpenoid biosynthesis-related genes [33]. Taken together, these data imply that the mechanisms of these terpenoid biosynthesis-related proteins in response to the present inhibitors might be different to the ones in response to ethanol.

Chemotaxis is a mechanism of bacteria responding to chemical stimulus. When exposed to chemicals, bacteria can move toward or opposite to them by the movement of flagella. However, chemotaxis proteins ZMO0082, ZMO0083, ZMO0085, ZMO0202, and CheB were down-regulated under the inhibitory conditions; in parallel, flagellum-related proteins FlgK, FlgI, FlgH, ZMO0611, FlhA, and FliS were also down-regulated (Additional file 4: Table S1). Similarly, transcriptional analysis of furfural-challenged *C. beijerinckii* [42] and *Z. mobilis* [33] showed that many genes related to chemotaxis and flagellar assembly were repressed. Under high-osmolality and low-pH conditions, the chemotaxis and flagellum-related proteins of *E. coli* were also down-regulated to reduce flagellum biosynthesis, thus restricting proton entrance during flagellum motor functioning [43]. In the energy consumption considerations, reducing flagellum biosynthesis is also a strategy to reserve the relatively expensive energy reserves for cells under detrimental environments [43].

Cell division is a way for bacteria to survive and maintain genetic information under harsh environment. FtsA is a cell division protein that promotes the formation of a circumferential structure, Z ring, which is crucial for cell division. It has been reported previously that overexpression of FtsA could help the colony formation and growth of *Streptococcus pneumoniae* in the absence of the KhpA/B RNA binding proteins by suppressing the requirement of PBP2b [44]. As FtsA was up-regulated in the presence of furfural, 5-HMF, and combined inhibitors, it is speculated that the up-regulation of FtsA might be helpful for *Z. mobilis* exposed to furans.

#### Energy production and conversion-related proteins

Transmembrane ATPases which catalyze the decomposition of ATP into ADP to release energy can help to import metabolites necessary for cell metabolism and export toxins, wastes, and solutes that can hinder cellular processes. ATP synthase family proteins like ATP12 ATPase, AtpH, AtpA, AtpG, AtpD, ZMO0242, and ZMO0915 were all up-regulated under the challenge of relevant inhibitors (Additional file 4: Table S1). Other proteins involved in the respiratory chain for energy production like type II NADH dehydrogenase Ndh; DSBA oxidoreductase ZMO0191; oxidoreductase ZMO1844; electron transfer flavoproteins ZMO1419, ZMO1480, and ZMO1842; glutathione synthetase GshB were also up-regulated. However, electron transfer flavoprotein dehydrogenase ZMO1184, pyrophosphate phospho-hydrolase Ppa, oxidoreductase FAD/NAD(P)-binding protein ZMO1753 were down-regulated. It has been reported previously that the loss of Ndh activity permits the acquisition of higher aerobic growth, enhanced ethanol production, and thermotolerance [45], while the down-regulation of Ndh was only detected for the cells in the presence of phenol. The up-regulation of Ndh in other groups implies that Ndh involved in different biological processes that respond to diverse inhibitors might have different roles to play. In fact, the complete physiological function of the *Z. mobilis* respiratory chain is still unknown up to some extent; although it is clear that the respiration in *Z. mobilis*, unlike in most facultative anaerobic and aerobic bacteria, does not produce energy source for aerobic growth [46].

#### DNA replication, recombination, repair, transcription, and RNA translation-related proteins

Replication, recombination, and repair of DNA are fundamental molecular mechanisms for organisms to maintain, regulate and evolve genetic information. The relevant proteins like excinuclease ABC subunits UvrA and UvrB, DNA repair protein RadA, 3′(2′),5′-bisphosphate nucleotidase ZMO0734, DNA polymerase III ZMO0980, Fapy-DNA glycosylase MutM, transcription-repair-coupling factor Mfd, and DNA mismatch repair protein MutS, primosomal protein PriA were all up-regulated in the relevant groups (Additional file 4: Table S1). However, S1/P1 nuclease ZMO0127, helicase domain protein ZMO0219, DNA polymerase I PolA, PepSY-associated TM helix domain protein ZMO0301, DNA topoisomerase IV subunit ParE, pyrimidine 5′-nucleotidase ZMO0430, peptidase U62 modulator of DNA gyrase ZMO1135, and DEAD/DEAH box helicase domain protein ZMO1417 were down-regulated. It is unclear that why recombinase RecA was up-regulated under the stress of phenol while down-regulated in the presence of formic acid, furfural, and combined inhibitors. Nevertheless, the regulation of these proteins, despite playing different roles in different biological processes, is helpful for cells to recover from the DNA damage caused by these inhibitors [47–49].

DNA is transcribed into RNA to transmit genetic information further, and during this step, transcriptional activators/repressors can regulate the transcription by promoting/blocking the recruitment of RNA polymerase to specific genes. As expected, in the current results (Additional file 4: Table S1), heat-inducible transcription repressor HrcA (ZMO0015), winged helix family proteins ZMO0257 and ZMO0478, RpsU-divergently transcribed protein ZMO0472, TetR family transcriptional regulator ZMO0963, Fis family transcriptional regulator ZMO1124, and LytTR family transcriptional regulator ZMO1738 were all up-regulated; while transcription termination proteins NusA and NusG, LysR family transcriptional regulator ZMO0781, ArsR family transcriptional regulator ZMO1748, and transcription termination factor Rho were down-regulated. Especially, as bacterial transcription initiation factors enable specific binding of RNA polymerase to gene promoters, sigma factors have been demonstrated to play a key role in resisting high ethanol and other stress conditions [34]. Accordingly, in the present results, sigma factors such as RpoN, FliA, RpoH, and ZMO0850 were up-regulated, while RpoB, RpoC, and RpoD were down-regulated, in response to the inhibitors.

After transcription, the translation process comprised of initiation, elongation, and termination is proceeded and conducted by ribosomes. In the mentioned groups of Additional file 4: Table S1, proteins related to translation, ribosomal structure and biogenesis such as ribosome hibernation promoting factor Hpf, GTPase HflX, ribonuclease R Rnr, and leucine-tRNA ligase LeuS were up-regulated, while elongation factors Efp and Tsf, translation initiation factor IF-2 InfB, and GTPase Era were down-regulated. Also, ribosomal proteins and aminoacyl-tRNA synthetases such as RpsG RplB, RplV, RpsC, RpsQ, RplN, RplE, RpsM, RimP, AspS, RplK, RplA, RplJ, GatB, RimO, ArgS, AlaS, RpsI, RplM, RlmN, MetG, RpmE, RplI, RpsD, RpsA, RplY, and RpsS were all down-regulated. It is clear that under the stress of these inhibitors, most proteins related to translation, ribosomal structure and biogenesis were of down-regulated expression pattern, which was in agreement with the transcriptomic results of furfural and acetate-challenged *Z. mobilis* [33, 39]. Down-regulation of these proteins, indicating an arrest of overall protein synthesis responsible for the diminished cell growth [33], might be partly caused by the fact that external stress can provoke mRNA degradation and inhibit translation process [50].

#### Posttranslational modification-, protein turnover-, chaperones-related proteins

Proteins related to posttranslational modification, protein turnover, and chaperones like stress shock-responsive molecular chaperone complex could respond to a variety of stress conditions, including extreme temperature, extreme cellular energy depletion, and extreme concentrations of ions and various toxic substances [51]. The molecular chaperone complexes such as ClpB, DnaK, GroES, GrpE, GroEL, DnaJ, and DnaJ domain protein ZMO1690 were all up-regulated in relevant groups (Additional file 4: Table S1); however, in previous studies, they were not significantly affected by the stress of ethanol [34]. All these proteins belong to a same stress-induced multi-chaperone system, which is important for the folding of newly synthesized polypeptides [52]. Furthermore, another chaperone protein HslU that is essential for the unfolding and translocation of proteins was also up-regulated. However, chaperone Tig which can maintain the newly synthesized secretory and non-secretory proteins in an open conformation was down-regulated in response to the mentioned inhibitors. Previous tests for disruption of gene-encoding proteins DnaK, GroEL, and HslU have confirmed that these proteins are necessary for normal growth of *E. coli* under detrimental environments like toxic antibiotics [53] and high temperature [54]. Therefore, regulation of these stress response molecular chaperones might be helpful for the acquirement of certain inhibitor tolerance of *Z. mobilis* cells.

Iron-sulfur (FeS) clusters are important cofactors for various proteins involved in electron transfer, in redox and non-redox catalysis, and in gene regulation. SufC ZMO0425 and SufBD ZMO0426, which constitute the SufBCD complex that can contribute to the assembly or repair of oxygen-labile FeS clusters under oxidative stress and the uptake of iron from extracellular iron chelators, were up-regulated (Additional file 4: Table S1). Furthermore, glutaredoxins ZMO0070 and ZMO0753, having roles in binding FeS clusters and delivering the clusters to specific enzymes on demand, were also up-regulated; they can also function as electron carriers and act in antioxidant defense in response to oxidative stress, using glutathione as a cofactor [55]. Similar to glutaredoxins, thioredoxin domain protein ZMO1705 sharing many of the functions of glutaredoxins mentioned above was also up-regulated within our expectations. Also, the up-regulated proteins related to glutathione included another two proteins, Prx ZMO1732 and glutathione S-transferase domain protein ZMO0935. Prx as an antioxidant can be reduced by thioredoxins or glutaredoxins, to keep cellular oxidant–antioxidant homeostasis; ZMO0935 is best known for its ability to conjugate xenobiotics to glutathione and thereby detoxify cellular environments.

Other proteins related to posttranslational modification like peptidases M28 family ZMO0145, M16 family ZMO1422, and M61 family ZMO1593, which can catalyze the hydrolysis of peptides into amino acids, were all down-regulated to regulate the protein degradation. Similarly, ZMO1593 was also down-regulated under the ethanol stress [10]. It is known that bacterial peptidases are involved in protein maturation, metabolism of peptides, and turnover of intracellular proteins, although little has thus far been known about their intrinsic functions [56, 57]. However, it implies that these peptidases are, perhaps, important in response to the present inhibitors.

#### Biosynthesis of amino acids-related proteins

Amino acids, as important primary metabolites involved in many biological processes, can help yeast cells to resist various inhibitors [58]. As expected, in the current study (Additional file 4: Table S1), many proteins related to biosynthesis of amino acids were differentially expressed, such as isopropylmalate isomerase LeuC, anthranilate phosphoribosyl-transferase TrpD, oligopeptidase ZMO0490, glutamine synthetase ZMO0493, *N*-(5′-phosphoribosyl) anthranilate isomerase TrpF, *O*-succinylhomoserine sulfhydryls MetZ, 3-isopropylmalate dehydrogenase LeuB, branched-chain amino acid aminotransferase ZMO0913, methionine synthase MetE, diaminopimelate epimerase DapF, acetolactate synthases ZMO1139 and ZMO1140, ketol-acid reductoisomerase IlvC, *N*-succinyl arginine dihydrolase AstB, serine methylase GlyA, threonine dehydratase ZMO1275, leucine aminopeptidase PepA, threonine aldolase ZMO1347, aspartate-semialdehyde dehydrogenase Asd, phosphoribosylformimino-5 -aminoimidazole carboxamide ribotide isomerase HisA, homoserine kinase ThrB, aminotransferase ZMO1682, phosphoserine aminotransferase ZMO1684, dihydroxy-acid dehydratase IlvD, threonine synthase ZMO1891, and methylated-DNA/protein-cysteine methyltransferase ZMO1989 were up-regulated. However, proteins like aromatic amino acid transaminase ZMO0937, peptidase S15 ZMO1167, cyclohexadienyl dehydrogenase TyrC, prolyl oligopeptidase ZMO0794, formate acetyltransferase ZMO1570, aspartokinase ZMO1653, and aconitate hydratase ZMO0543 were down-regulated. Arginine, serine, histidine, and aromatic amino acids can significantly enhance the tolerance of *E. coli* to furfural [59], and arginine and lysine can also improve the tolerance of *Salmonella typhimurium* to acetic acid [60]. However, the up-regulation of proteins related to arginine, histidine, and lysine were not found in this study except for the serine-related protein GlyA, implying that discrepancies of the tolerance to certain inhibitors exist in different microorganisms.

#### Central carbon metabolism-related proteins

Carbon metabolism is the most basic aspect of living beings. In central carbon metabolism pathway, up-regulated proteins involved in ED and TCA cycle pathways mainly included glyceraldehyde 3-phosphate dehydrogenase GAP, phosphoglycerate kinase PGK, fructose-bisphosphate aldolase FBP, glucokinase GLK, E3 subunit α-ketoglutarate malate dehydrogenase KDHC, isocitrate dehydrogenase ICDH, 2-keto-3-deoxy-phosphogluconate aldolase EDA, phosphoglucose isomerase PGI, alcohol dehydrogenase I ADHA, enolase ENO, malic enzyme ME, and citrate synthase CS (Additional file 4: Table S1). Other relevant proteins like glucose 6-phosphate dehydrogenase ZWF, 6-phosphogluconate dehydratase EDD, phosphoglyceromutase PGM, phosphogluconolactonase PGL, and phosphoenolpyruvate carboxylase PPC were also up-regulated, in most cases. However, ZWF in group F; EDD, fumarase FUM, and PPC in group H; AH in groups P and C; PGL and PGM in group P were down-regulated. The different expression patterns of these proteins illustrated that carbon fluxes in certain metabolic pathways were quite complex in response to various external stress. Previously, it was also revealed that many proteins related to carbohydrate metabolism were differentially expressed in response to high concentration of glucose [32], phenolic aldehydes [22], furfural [33], and ethanol [34], although these results are not entirely the same with ours.

### Metabolomic analysis combined with proteomics

Cellular glucose was detected to be up-regulated in groups FA and AA (Table 1), which implies that an acidic environment may affect the uptake and consumption of glucose. Before being phosphorylated, glucose can be transported across the plasma membrane by a facilitated diffusion system [61], and afterward catalyzed to gluconic lactone by certain enzymes. Further, gluconic lactone can be catalyzed to gluconic acid by gluconolactonase ZMO1649, although this enzyme was not identified in our proteomics results. Under weak acid stress, cells need to adjust the intracellular level of H^+^ to keep H^+^ balance. Thus, as shown in Table 1, gluconic lactone which can cause the drop of pH [62] was up-regulated in response to formic acid; gluconic lactone was also up-regulated under the acetic acid stress, although it was ruled out statistically by our OPLS-DA analysis. Furthermore, gluconic acid as an acidity regulator that can help change or maintain pH was also up-regulated under the acidic stress. In addition, gluconic lactone and gluconic acid might also play roles in resisting other inhibitors like furfural, 5-HMF, and phenol, although their expression patterns were different in response to different inhibitors (Table 1).

Phenylalanine can be formed from phenylpyruvate by the catalyzation of ZMO0937, which can catalyze 4-hydroxyphenyl pyruvate to tyrosine, too. Besides, tyrosine can also be formed from l-arogenate by the catalyzation of ZMO0420. Thus, the down-regulation of ZMO0937 and ZMO0420 can lead to a decrease of phenylalanine and tyrosine, which was verified by our results of groups FA, AA, F, and H (Table 1 and Additional file 4: Table S1). Alanine and aspartate can interconvert into each other by the catalyzation of aminotransferase classes I and II ZMO1682. Hence, the up-regulation of ZMO1682 along with the down-regulation of aspartic acid in groups F, H, and P and the up-regulation of alanine in group C were within our expectations. However, the up-regulation of ZMO1682 resulted in down-regulation of alanine in group FA, which might be owing to that alanine was converted into pyruvate and further flowed into other metabolic pathways under the acidic stress.

Leucine in groups F and C; isoleucine in groups AA, F, H, P, and C; valine in groups AA, F, H, and P were all up-regulated (Table 1). In bacteria, leucine, isoleucine, and valine are synthesized from pyruvate employing the same enzymes [63], namely LeuC, LeuB, ZMO0687, ZMO1139, ZMO1140, IlvC, IlvD, ZMO0115, and ZMO0913. These enzymes were all up-regulated in our proteomics results by the up-regulation of metabolites leucine, isoleucine, and valine, in most cases (Table 1 and Additional file 4: Table S1). The exception was the down-regulation of leucine in group FA. Leucine and 2-oxopentanoate can interconvert into each other, catalyzed by bi-directional enzymes ZMO0115 and ZMO0913. Thus, the up-regulation of ZMO0115 and ZMO0913 leading to the down-regulation of leucine was reasonable.

Serine and glycine can interconvert with each other by the catalyzation of bi-functional glycine hydroxymethyltransferase GlyA, and so can glycine and l-allothreonine by the catalyzation of threonine aldolase ZMO1347. Furthermore, l-allothreonine can be converted from O-phospho-l-homoserine by threonine synthase ZMO1891. Accordingly, the up-regulation of proteins GlyA, ZMO1891, and ZMO1347 can further lead to the up-regulation of metabolites serine, glycine, and l-allothreonine; this was consistent with most of our results in groups AA, F, H, P, and C, except that l-allothreonine in groups AA and F was down-regulated (Table 1 and Additional file 4: Table S1). It implies that l-allothreonine was also affected by other unknown factors in groups AA and F. Also, proline can be transformed from (S)-1-pyrroline-5-carboxylate by the enzyme pyrroline-5-carboxylate reductase ProC (ZMO0311). Correspondingly, proline and ProC were both up-regulated in groups AA, F, H, P, and C, regardless of the *p* values of ProC which were more than 0.05. Besides, the formation of *O*-Phosphoserine can be positively catalyzed by phosphoserine aminotransferase ZMO1684; hence, it is within our expectations that *O*-Phosphoserine and ZMO1684 were both up-regulated in the FA group.

Fatty acids like linoleic acid methyl ester, stearic acid, and oleic acid are essential components of membranes and important to maintain membrane lipid homeostasis [64]. Thus, up-regulation of these fatty acids in groups AA, F, H, and C to resist the inhibitors was within our expectations (Table 1). Besides, succinic acid can be transformed from fumarate by succinate dehydrogenase ZMO0569 and transformed from succinyl-CoA by succinyl-CoA synthetase ZMO0567 and Succinyl-CoA ligase SucC ZMO1481, while it also can be transformed from succinate semialdehyde by aldehyde dehydrogenase ZMO1754. There were no significant differences in ZMO0569, ZMO0567, and ZMO1481; whereas, the highly up-regulated ZMO1754 could interpret the up-regulation of succinic acid in groups AA, F, H, and C, with an exception that succinic acid in group P was up-regulated while none of the relevant enzymes was up-regulated. Another weak acid, lactic acid is transformed from part of pyruvic acid to generate ATP by d-lactate dehydrogenase ZMO0256. However, no significant differential expression of ZMO0256 was detected, while the down-regulation of lactic acid in groups FA and P might result from the adjustments of other biological processes.

Phosphorylation and dephosphorylation, which are important pathways for cells to store and release energy, are pivotal in the regulation of metabolic processes. Correspondingly, phosphates were down-regulated in groups AA, F, and H in response to the stress of inhibitors (Table 1). Furthermore, trehalose is synthesized specially upon different stresses to protect various cellular components, and it can also directly interact with nucleic acids to facilitate melting of double-stranded DNA and stabilize single-stranded nucleic acids [65]. Hence, the up-regulation of trehalose in groups F, P, and C were consistent with its positive effects. However, why trehalose was down-regulated in group FA is unknown. Besides, ribosomal RNA and ribosomal proteins together constitute functional ribosomes. Accordingly, as the two parts of the ribosome, ribosomal protein and ribosomal RNA should be changed in the same manner. In the present results, the down-regulation of ribosomal RNA related ribose in groups FA, AA, F, and H was in agreement with the down-regulation of most ribosomal proteins (Table 1 and Additional file 4: Table S1).

To the best of our knowledge, some metabolites like oxoproline, glycerol, d-glyceric acid, gluconic lactone, inosine, dihydroxyacetone, 1, 2-cyclohexanedione, 3-hydroxypropionic acid, and 2-hydroxypyridine are involved in KEGG pathways that no enzymes are annotated in the database to facilitate these pathways. Therefore, the proteins directly correlated with these metabolites are not analyzed and presented here.

### Universal biomarker proteins and metabolites shared by the six inhibitor-treated groups

**Fig. 3:**
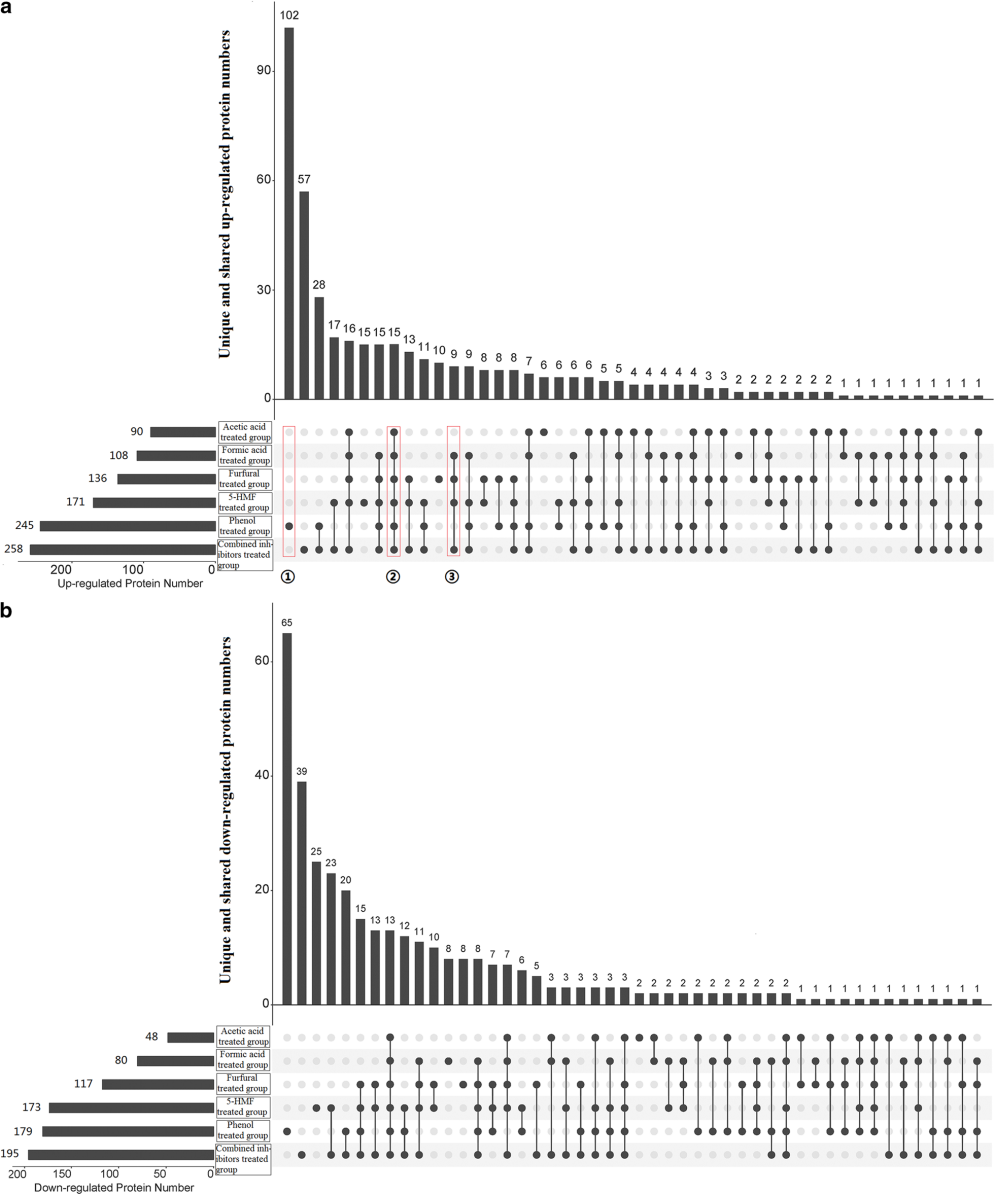
Numbers of the unique and shared differentially expressed proteins of the six inhibitor-treated groups. **a** The numbers of unique and shared up-regulated proteins in the presence of different inhibitors, and **b** the numbers of unique and shared down-regulated proteins in the presence of different inhibitors. These protein numbers are shown in an UpSet diagram, which allows for a clearer plotting of large data sets compared to the Venn diagram. Dark circles connected with a line indicate that the proteins are only differentially expressed in the corresponding groups labeled with dark circles, but not in the other groups labeled with gray circles. For example, ① denotes the proteins are only differentially expressed in phenol-treated group, while not differentially expressed in the other groups, that is, these proteins are unique in phenol-treated group; ② denotes the proteins are differentially expressed in all the groups, that is, these proteins are shared by all the groups; ③ denotes the proteins are only differentially expressed in formic acid, furfural, 5-HMF, and combined inhibitors treated groups, while not differentially expressed in acetic acid and phenol-treated groups. Combined inhibitors means a combination of formic acid, acetic acid, furfural, 5-HMF, and phenol

Before the overall comparison of the six inhibitor-treated groups, biomarker proteins and metabolites caused by similar inhibitors were compared. Our proteomics and metabolomics profiles of groups FA and AA showed that 89 proteins (counted according to the unique and shared DEP numbers shown in Fig. 3) and 11 metabolites (Table 1) were simultaneously differentially expressed in both groups. The high proportion of collectively shared proteins and metabolites between these two groups illustrated that formic acid and acetic acid probably showed similar effects on *Z. mobilis* ZM4, although each group also had its unique proteins and metabolites. In fact, it has been reported that undissociated weak acids like formic acid and acetic acid can both cause the differential expression of proteins related to biosynthesis of amino acids, energy, and oxidoreductase in many microorganisms [58].

Furaldehydes like furfural and 5-HMF exerted similar effects on *Thermoanaerobacter pseudethanolicus* [48] and *S. cerevisiae* [2, 66], by reducing the expression of proteins related to protein synthesis, glycolytic pathway, TCA cycle, sulfur amino acid biosynthesis, cellular signaling pathways, and heat shock proteins. Similarly, it is shown that 192 proteins and 17 metabolites (Fig. 3 and Table 1), which both took up high proportions of the total numbers in relevant groups, were simultaneously differentially expressed in groups F and H. Further, the DEPs were related to many aspects of cellular biological processes and functions, which showed high levels of consistency with the previous transcriptome results [33] of *Z. mobilis* ZM4 treated by furfural.

Phenolic compounds are also able to induce loss of cell membrane integrity and accumulation of ROS [66]. Transcriptome analysis showed that genes encoding certain reductases and transporter proteins were responsible for the resistance of phenolic aldehydes [22]. In our results, significantly differential expression of many reductases and transporter proteins, including the key ones for phenolic aldehydes resistance like ZMO0282 and ZMO0283 revealed in the literature [22], were also exhibited. Especially, the number of unique DEPs in group P far outstripped the ones in the other groups (Fig. 3), illustrating that the mechanism of *Z. mobilis* ZM4 in response to phenol was probably different from that responding to the other inhibitors.

Furthermore, the proteomics and metabolomics analysis of *Z. mobilis* ZM4 under the challenge of combined inhibitors showed that nearly all of the biological processes and metabolic pathways were synergistically affected by the combined inhibitors, as much higher fold changes of most differential proteins and metabolites were obtained here than that obtained under the challenge of a single inhibitor. Nevertheless, deep analysis is also needed to reveal how *Z. mobilis* responds to these combined inhibitors and what common information underlies these results.

**Fig. 4:**
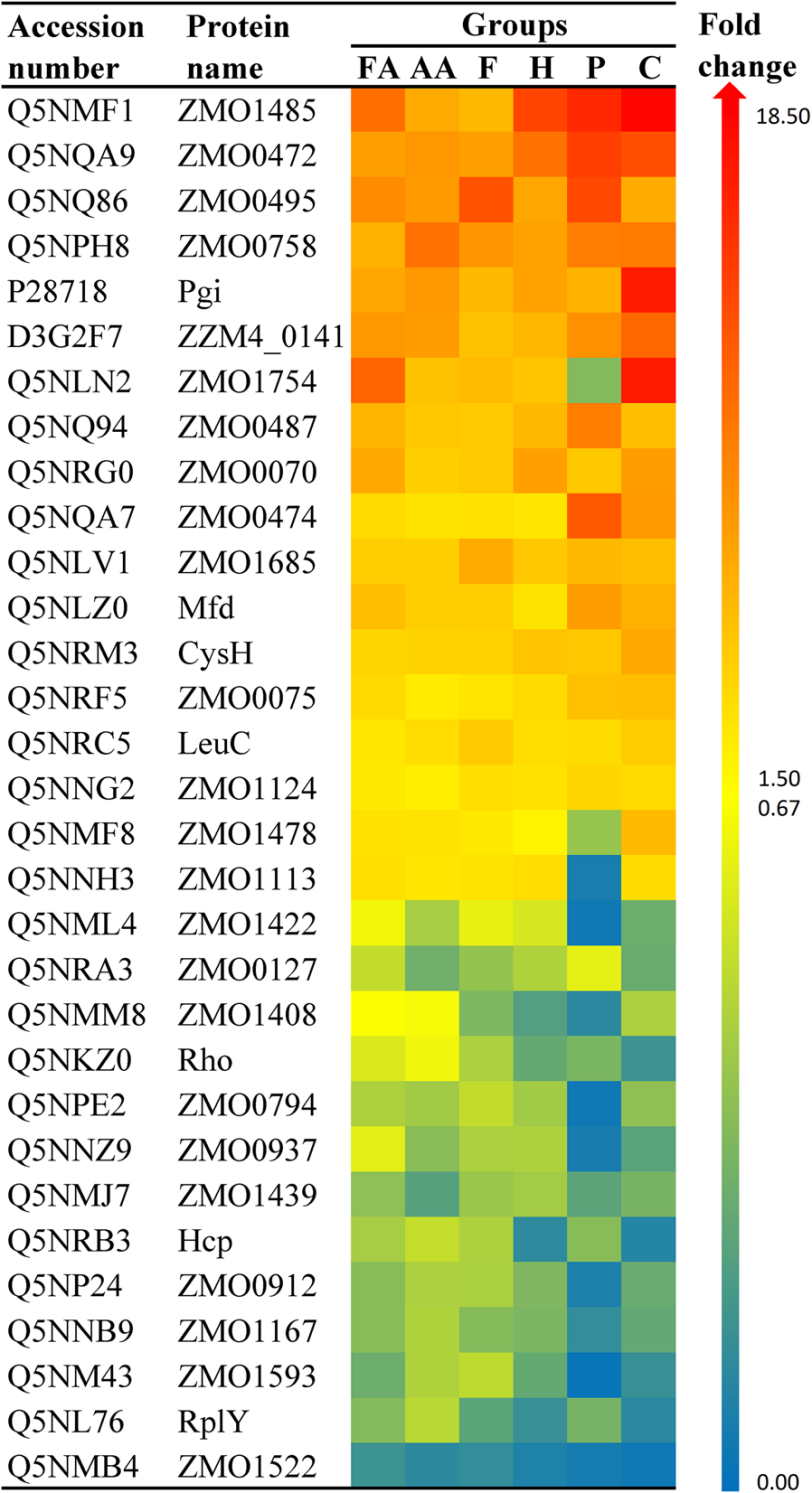
Heat map of the differentially expressed proteins shared by the six inhibitor-treated groups. Rows are colored by the fold changes of the proteins in inhibitor-treated groups relative to the corresponding proteins in control. *FA* cells treated by formic acid, *AA* cells treated by acetic acid, *F* cells treated by furfural, *H* cells treated by 5-HMF, *P* cells treated by phenol, *C* cells treated by combined inhibitors. The darker the red color, the greater the up-regulation fold change. The darker the blue color, the greater the down-regulation fold change

To reveal the universal mechanism of *Z. mobilis* ZM4 in response to combined inhibitors, collectively shared differential proteins and metabolites among all these groups were the focus of our attention. Total 31 DEPs were simultaneously present in all the groups (Fig. 4), which means that these DEPs are candidate proteins to help *Z. mobilis* ZM4 cells resist the adverse environment caused by the biomass-derived inhibitors. Of the 31 DEPs, 15 DEPs (ZMO1485, ZZM4_0141, ZMO0495, ZMO0472, Pgi, ZMO0070, ZMO0758, ZMO0487, Mfd, ZMO1685, CysH, ZMO0075, ZMO0474, LeuC, and ZMO1124) were simultaneously up-regulated; wherein, ZMO1485 showed the highest fold change with an average value of 11.95, followed by ZMO0472 with average 10.25-fold change and ZMO0495 with average 9.53-fold change (Fig. 4). Furthermore, 13 DEPs (ZMO1408, ZMO1422, ZMO0937, Rho, ZMO0127, ZMO0794, Hcp, ZMO1439, ZMO1167, ZMO0912, RplY, ZMO1593, and ZMO1522) were simultaneously down-regulated; wherein, ZMO1522 exhibited the highest fold change with an average value of 0.10, followed by RplY with average 0.27-fold change and ZMO1593 with average 0.28-fold change (Fig. 4). Also, the metabolic pathways and Gene Ontology categories that these DEPs were mainly involved in are presented in Additional file 1: Fig. S5, in which it is shown that these pathways and categories were pretty diverse. The protein–protein interactions of these DEPs are also shown in Additional file 1: Fig. S6, wherein it can be seen that PGI, RplY, and LeuC have the maximal interaction numbers with other DEPs, followed by GabD, CysH, and ZMO1685. Here, protein–protein interactions are determined if the two proteins have been documented to be neighborhood proteins, fusion proteins, co-expression proteins, etc. with a confidence score more than 0.40.

To get a better insight, these DEPs were further categorized into several aspects according to Cluster of Orthologous Groups of proteins (Table 2). Leucine- and isoleucine biosynthesis-related isopropylmalate isomerase LeuC, tyrosine- and phenylalanine biosynthesis-related transaminase ZMO0937, and protein degradation-related peptidases ZMO1408, ZMO0794, and ZMO1167 are involved in amino acid transport and metabolism (Table 2). LeuC could bind to FeS clusters that are involved in electron transfer and redox reaction, and in *E. coli* it was proved that overexpression of LeuC could partially relieve the growth defect caused by the oxidative stress of H_2_O_2_ [67]. Thus, up-regulation of LeuC would be helpful for *Z. mobilis* tolerant to the inhibitors. Although it is still not clear how peptidases ZMO1408, ZMO0794, and ZMO1167 help cells to resist the inhibitors, it is known that peptidases can no longer simply be thought of as essential for protein degradation and recycling, because they are also vital regulators and signaling molecules [68].

**Table 2:** Functional categories of the biomarker proteins shared among all the groups

Biomarker proteins	Functional categories
LeuC, ZMO1408, ZMO0937, ZMO0794, ZMO1167	Amino acid transport and metabolism
ZMO0758, ZMO0487, ZMO1685, CysH, ZMO0474	Coenzyme transport and metabolism
ZMO0070, ZMO1422, ZMO1593	Posttranslational modification, protein turnover, and chaperones
ZMO0472, ZMO1124, Rho	Transcription
Pgi, ZMO1478	Carbohydrate transport and metabolism
Mfd, ZMO0127	Replication, recombination, and repair
ZMO1439, ZMO1522	Cell wall/membrane/envelope biogenesis
ZMO0495, ZMO0912	Uncharacterized protein
ZMO1754, ZMO1113	Energy production and conversion
ZMO1485	Nucleotide transport and metabolism
RplY	Translation, ribosomal structure and biogenesis
Hcp	Inorganic ion transport and metabolism
ZMO0075	Signal transduction mechanisms
ZZM4_0141	General function prediction only

Inhibitors can cause DNA damage [47–49]. Hence, the up-regulated transcription-repair-coupling factor Mfd that can couple transcription and DNA repair by recognizing RNA polymerase stalled at DNA lesions is significant for DNA replication, recombination, and repair. It was reported that the interaction of Mfd with UvrA could enhance the rate of DNA repair [69]; accordingly, to help Mfd repair the impaired DNA, UvrA might also be up-regulated, which was verified by our results in most cases (Additional file 4: Table S1). Another DNA degradation- and repair-related protein, S1/P1 nuclease ZMO0127, which can cleave RNA and single-stranded DNA to mononucleosides was down-regulated (Table 2). Although its primary substrate is single-stranded DNA, S1/P1 nuclease can also occasionally introduce single-stranded breaks in double-stranded DNA [70]. Thus, down-regulation of ZMO0127 might be protective toward normal DNA, at least to some extent.

Further, transcriptional regulators ZMO0472, ZMO1124, and Rho (Table 2) play a critical role in transcription. Especially, ZMO1124 is a Fis family transcriptional regulator; in *Acinetobacter*, this regulator was determined to have a role in regulating the expression of genes related to biodegradation of phenol inhibitor [71]. Also, it has been reported previously that the inhibition of Rho function could help *E. coli* to survive under oxidative stress [72]. Therefore, regulation of these transcriptional regulators might be important for cells to overcome the inhibitory environment.

Translation-related 50S ribosomal protein L25 RplY, also known as general stress protein CTC, which can be induced by stress condition, is a general stress protein of *E. coli* and *Bacillus subtilis* [73]. However, in the present results, it was down-regulated under the challenge of inhibitors (Table 2), which implies that the regulatory mechanism of RplY in *Z. mobilis* might be different from those in other microorganisms. Besides, three proteins including glutaredoxin ZMO0070 and peptidases ZMO1422 and ZMO1593 are involved in posttranslational modification. ZMO0070, as a ubiquitous small heat-stable oxidoreductase, functions in many cellular processes like deoxyribonucleotide synthesis, reparation of oxidatively damaged proteins, proteins folding, and sulfur metabolism [74]; therefore, the up-regulation of ZMO0070 (Table 2) would be helpful for cells to resist inhibitors. Peptidases ZMO1422 and ZMO1593 were down-regulated herein, similar to the above-mentioned amino acid transport and metabolism-related peptidases (Table 2). Previously, Peptidase ZMO1593 was also found to be down-regulated under ethanol stress [10]. It is known that bacterial peptidases lay physiological functions not only in generalized protein degradation but also in protein maturation, metabolism of peptides, and turnover of intracellular proteins; however, little is known about their specific functions [56, 57], especially the function on stress response.

It is also notable that the nucleotide transport and metabolism-related deoxyguanosine triphosphate triphosphohydrolase-like protein ZMO1485 was up-regulated under all the stress conditions (Table 2). ZMO1485 is a dGTPase that can hydrolyze dGTP to constituent deoxynucleoside and inorganic triphosphate, leading to the decrease of dGTP and restriction of DNA and RNA synthesis. However, this is beneficial for microbial cells to prevent DNA mutagenesis [75, 76], and it also might be a way for cells to prevent unnecessary DNA and RNA synthesis to reserve energy under inhibitory environment. What is unexpected was that energy-related aldehyde dehydrogenase ZMO1754 responsible for the oxidation of aldehydes to carboxylic acids and Ndh responsible for the electron transfer were down-regulated in the phenol-treated group while up-regulated in the other groups (Table 2), which elucidate that these two proteins responded to phenol and the other inhibitors with different patterns.

Apolipoprotein *N*-acyltransferase ZMO1439 and TonB-dependent transporter ZMO1522 are proteins related to cell wall/membrane/envelope biogenesis. ZMO1439 which is responsible for the *N*-acylation of apolipoprotein in lipoprotein maturation is important for lipoproteins transport from the plasma to the outer membrane, and further, depletion of apolipoprotein *N*-acyltransferase could result in increased outer membrane permeability [77]. However, in this study, why apolipoprotein *N*-acyltransferase was down-regulated is not clear. Provided that *Z. mobilis* 8b could adjust itself to reduce energy needs for substrate uptake and reserve energy for stress responses via down-regulating the outer membrane transport protein ZMO1522 [39], it is speculated that, although required a further verification, down-regulation of apolipoprotein *N*-acyltransferase might also play a role in reducing energy expenditure. Hence, the down-regulation of membrane transport proteins like ZMO1522 might provide some benefit for the survival of *Z. mobilis* cells. Also, signal transduction-related outer membrane protein ZMO0075, a PhoH family phosphate starvation-inducible protein, was up-regulated (Table 2). Although PhoH-like phosphate starvation-inducible protein of *Prochlorococcus* could respond to low nutrient condition [78], it is still necessary to elucidate the biological function of ZMO0075 in *Z. mobilis* comprehensively. Similarly, uncharacterized proteins ZMO0495 and ZMO0912 (Table 2) shared by cells under all the stress conditions also required further studies to reveal their potential roles in resisting inhibitors, although they have been structurally predicted to possess transmembrane helix domain and coiled-coil domain, respectively.

Inorganic ion transport and metabolism-related hybrid cluster protein Hcp was down-regulated in this study (Table 2). Although it generally has a role in the detoxification of toxic nitrogen compounds [79, 80], it was also reported that in different microorganisms it has distinct physiological functions like resistance to oxidative stress [80]. To our knowledge, what the down-regulation of Hcp implies to *Z. mobilis* in stress conditions is still on the way to be clarified.

Additionally, five proteins (ZMO1685, CysH, ZMO0758, ZMO0487, and ZMO0474) related to coenzyme transport and metabolism shown in Table 2 were all up-regulated. d-3-phosphoglycerate dehydrogenase ZMO1685 as one of the important enzymes involved in the glycolytic pathway can catalyze the transition of 3-phosphoglycerate into 3-phosphohydroxypyruvate and produce NADH, which is the committed step in the phosphorylated pathway of l-serine biosynthesis. Excess NADH can be partly consumed by the formation of glycerol to maintain the intracellular redox balance [81]. Therefore, the glycerate pathway-related protein ZMO1685 might play a role in resisting inhibitors via regulating amino acids biosynthesis and redox balance. Phosphoadenosine phosphosulfate reductase CysH that catalyzes the reduction of activated sulfate into sulfite is controlled in response to sulphur/cysteine availability by the CysB transcriptional regulator, which also plays roles in resistance to oxidative stress and antibiotic susceptibility in *E. coli* [82]; it means that the sulfur metabolism pathway connected to other global processes [82] might also be important for microbial cells to resist adverse environment. Besides, it is known that isochorismatase ZMO0758 can hydrolyze isochorismate into pyruvate and 2,3-dihydroxy-2,3-dihydroxybenzoate, HpcH/HpaI aldolase ZMO0487 can convert 4-hydroxy-2-oxoheptanedioate into pyruvate and succinate semialdehyde, and 3,4-dihydroxy-2-butanone 4-phosphate synthase ZMO0474 can catalyze the conversion of d-ribulose-5-phosphate into formate and 3,4-dihydroxy-2-butanone-4-phosphate. Although these three proteins can affect the carbohydrate metabolism process, how they help *Z. mobilis* to resist stress conditions is unclear now.

**Fig. 5:**
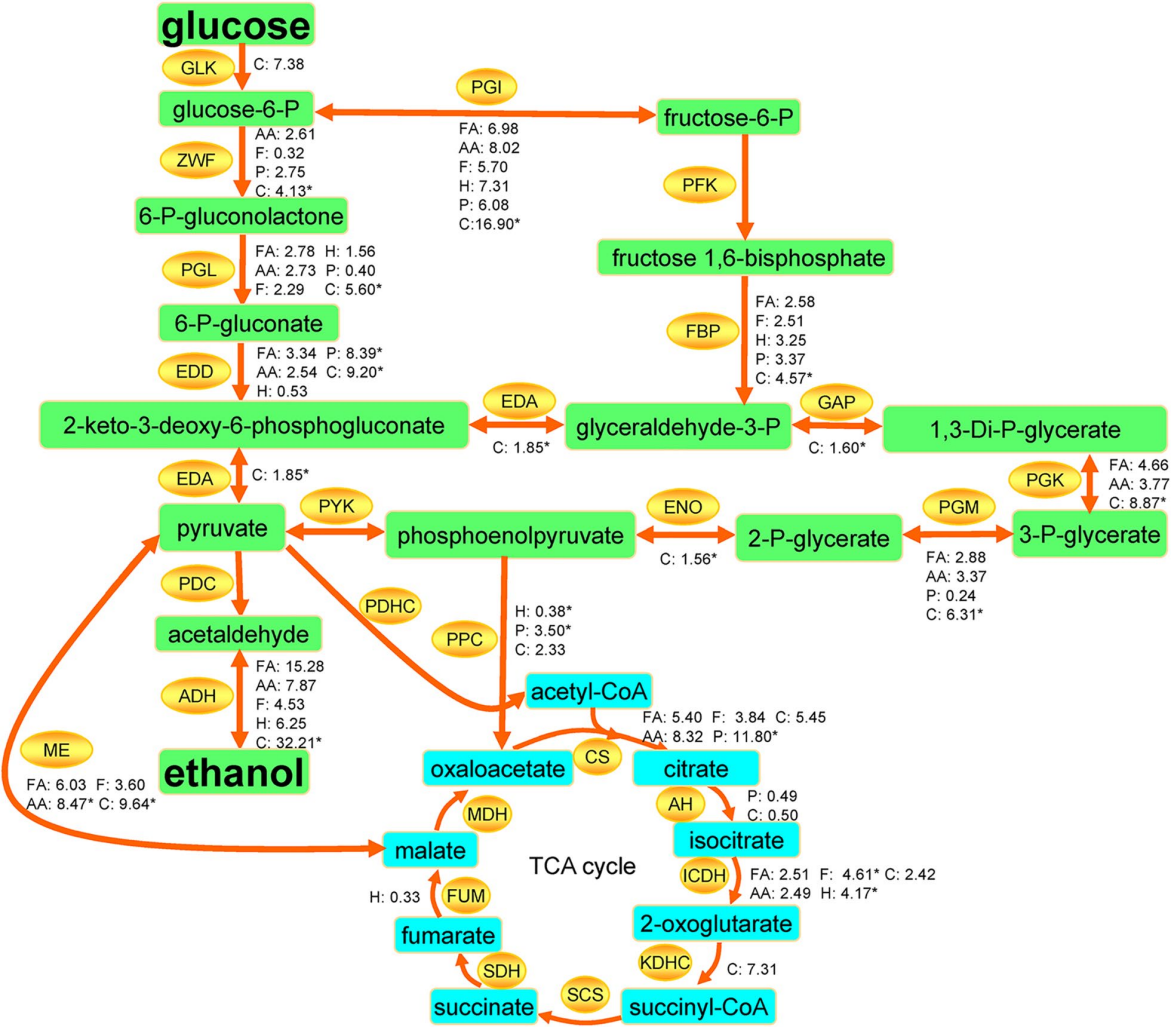
Influences of different inhibitors on the enzymes involved in central carbon metabolism. *GLK* glucokinase, *PGI* phosphoglucose isomerase, *ZWF* glucose 6-phosphate dehydrogenase, *PGL* phosphogluconolactonase, *EDD* 6-phosphogluconate dehydratase, *PFK* phosphofructokinase, *FBP* fructose-bisphosphate aldolase, *GAP* glyceraldehyde 3-phosphate dehydrogenase, *EDA* 2-keto-3-deoxy-phosphogluconate aldolase, *PGK* phosphoglycerate kinase, *PGM* phosphoglyceromutase, *ENO* enolase, *PYK* pyruvate kinase, *PDC* pyruvate decarboxylase, *ADHA* alcohol dehydrogenase I, *ME* malic enzyme, *PDHC* pyruvate dehydrogenase complex, *PPC* phosphoenolpyruvate carboxylase, *CS* citrate synthase, *AH* aconitate hydratase, *ICDH* isocitrate dehydrogenase, *KDHC* E3 subunit α-ketoglutarate malate dehydrogenase, *SCS* succinyl-CoA synthetase, *SDH* succinate dehydrogenase, *FUM* fumarase, *MDH* malate dehydrogenase. Fold-change values of the enzymes under the challenge of different inhibitors are marked beside the enzyme names. Symbol “*” denotes *p* value < 0.0001

In addition, two glycolytic enzymes related to carbohydrate transport and metabolism, PGI and PGL, were also up-regulated under the stress of these inhibitors, except PGL in the phenol-challenged group. In fact, how the inhibitors affect central carbon metabolism is another issue of concern, because central carbon metabolism is not only related to ethanol production, the end-product of interest, but also related to the redox balance and energy supply for cells. In our results, most key enzymes involved in ED pathway and pyruvates biosynthetic pathway like ZWF, PGI, PGL, EDD, FBP, PGK, PGM, and ADHA were up-regulated under the challenge of these inhibitors (Fig. 5), and so were the enzymes involved in TCA cycle like PPC, ME, CS, and ICDH. These results are in line with the proteomic analysis of ethanol-challenged *Z. mobilis* that most proteins involved in the ED pathway and energy metabolism were up-regulated [10]. Enzymes GLK, ZWF, and EDD are derived from the same operon that controls glucose uptake and the initial steps of glucose metabolism, which enables rapid glucose utilization [83]. Other enzymes shown in Fig. 5 could also affect glucose metabolism, thus affecting the redox balance and energy supply for cells. For example, ADHA, as the prominent enzyme with highest fold change among these enzymes, is important in the central carbon metabolism pathway; it not only can reduce acetaldehyde to ethanol and facilitate the regeneration of NAD^+^ for energy-generating glycolysis to continue, but also can help cells to resist oxidation stress [84]. In fact, there already existed evidence that ADHA of a *Z. mobilis* strain obtained by adaptive evolution was up-regulated in response to furfural and acetic acid [14]. The up-regulation of these enzymes related to central carbon metabolism implied that these enzymes could, in some extent, contribute to the recovery of cell growth from stress conditions by improving energy supply and regulating redox equilibrium, although the cell growth rates under these inhibitors were still much lower than that in the absence of the inhibitors. Notably, PGI that can interconvert glucose-6-phosphate and fructose-6-phosphate was up-regulated under all the stress conditions. Mutation of PGI-encoding gene *pgi* in *E. coli* could disturb the balance of cellular NADPH [85] and lead to the down-expression of several anaerobically induced genes and some supercoiling-dependent promoters [86, 87], which proved that PGI plays important roles in the osmotic and anaerobic responses and the regulation of stress-regulated promoters of *E. coli* [86]. Thus, it is concluded that regulation of these enzymes like PGI, could probably enhance glucose metabolism, increase energy supply, regulate redox equilibrium, and provide metabolic intermediates for other biological processes to resist adverse environment.

Regarding the biomarker metabolites, only oxoproline and gluconic acid were simultaneously present in all the groups, however, they changed with different regulation patterns in different groups (Table 1). Interestingly, in group C, all the biomarker metabolites were up-regulated, which means that combination of these five inhibitors exerted synergistic effects on amino acids and central carbon metabolism intermediates when compared to the results obtained in groups treated by the single inhibitor. Unlike the expression of one protein is conducted by one gene, one metabolite can be involved in many metabolic pathways and is thus affected by various factors. Therefore, most metabolites under the challenge of different inhibitors might not exhibit consistent change rules. However, as shown in the previous section, metabolomics results could support complementary data for proteomics results to elucidate much about how these inhibitors exert adverse effects on *Z. mobilis* cells and how *Z. mobilis* in return responds to these inhibitors.

## Conclusion

Overall, proteomics and metabolomics together provided insight into the cellular responses of *Z. mobilis* ZM4 under the stress of different inhibitors, which indicates that tolerance of *Z. mobilis* ZM4 to the various biomass-derived inhibitors is acquired by modulating various biological processes that take place at cellular levels. In this study, proteins related to cell wall/membrane biogenesis, cell motility, energy production, DNA replication, DNA recombination, DNA repair, DNA transcription, RNA translation, posttranslational modification, biosynthesis of amino acids, and central carbon metabolism were differentially expressed. Particularly, 15 up-regulated proteins (ZMO1485, ZMO0495, ZMO0472, PGI, ZMO0070, ZMO0758, ZMO0487, Mfd, ZMO1685, CysH, ZMO0075, ZMO0474, LeuC, and ZMO1124) and 13 down-regulated proteins (ZMO1408, ZMO1422, ZMO0937, Rho, ZMO0127, ZMO0794, Hcp, ZMO1439, ZMO1167, ZMO0912, RplY, ZMO1593, and ZMO1522) were further selected as candidate biomarkers responsible for inhibitor tolerance. It could be concluded that regulating some of these proteins (e.g., LeuC, Mfd, PGI, ZMO1522, and Rho) might be helpful for *Z. mobilis* ZM4 to successfully survive under the stress of various inhibitors and convert the biomass-derived substrates into valuable chemicals. The results from this study could improve our understanding of bacterial tolerance to complex inhibitors. Furthermore, this information can also be used to generate a database, which will facilitate the development of inhibitor-resistant strains for efficient fermentation processes. Further work about overexpression, suppression, or knockout of the relevant genes corresponding to the presently reported biomarkers by genetic manipulation is required to develop robust strains of *Z. mobilis* resistant to multiple inhibitors in the industrial applications.

## Methods

### Microorganism and culture conditions

The *Z. mobilis* strain ZM4 (China Center of Industrial Culture Collection, CICC 10273) was used in this study. T medium composed of (w/v) 2% glucose, 1% yeast extract, 0.05% MgSO_4_, 0.1% (NH_4_)_2_SO_4_, and 0.1% KH_2_PO_4_ was used for liquid culture of *Z. mobilis*, and solid T medium with 1.8% agar was used for strain maintenance. The first-grade seed culture was prepared by transferring a single colony of *Z. mobilis* strain into 100 mL autoclaved T medium in a 250-mL Erlenmeyer flask. The flasks were sealed with a rubber stopper and kept in a shaker at 30 °C and 150 rpm for 24 h. A second-grade seed culture was prepared by transferring 1% (v/v) of the first-grade seed culture into 100 mL fresh T medium, and finally, the seed culture with a cell density (~ 2 × 10^7^ cells/mL) was used for subsequent inoculation experiments.

### Cell growth under different concentrations of the inhibitors

The inhibitory effects of the representative inhibitors (Formic acid, acetic acid, furfural, 5-HMF, and phenol) on cell growth were determined by adding different concentrations of each inhibitor to the autoclaved T medium before inoculation. Culture medium without inhibitors was set as control. Cells were grown simultaneously in the presence or absence of inhibitors and sampled at the same time post-inoculation. OD_600_ values representing discrepancies of cell growth were used to show the effects of different inhibitors. In the preliminary experiments, increasing concentrations of each inhibitor with an interval of 0.5 g/L were first added to the culture media to determine the minimum inhibitory concentration (MIC) of each inhibitor. Based on the data obtained, the concentration of each inhibitor with an increment of 0.1 g/L was further tested to precisely determine the MIC of these inhibitors. Finally, a series of combinations of all the inhibitors containing 0.5% to 100% of the MIC of each inhibitor, that is, 0.5% to 100% of the combination of the MIC (CMIC), was used to challenge *Z. mobilis* cells. Three biological replicates were independently established for each experiment. Based on the results obtained, appropriate concentrations of each inhibitor and their combination were used in the next steps to prepare samples and perform proteomics and metabolomics analysis.

### Determination of cell growth, glucose and ethanol concentrations

Optical densities of each sample were measured using a Unico UV-4802 spectrophotometer (Unico Instrument Co., Ltd., Shanghai, China). Analyses of glucose and ethanol were performed on a high-performance liquid chromatography system (HPLC, LC-20AT, Shimadzu Corporation) equipped with a RID-10A refractive index detector and a HCT-360 column heater. The analytical column used was a Transgenomic ICSep ICE-ION-300 column (7.8 mm × 300 mm). The mobile phase was 0.00085 N sulfuric acid at a flow rate of 0.4 mL/min, the injection volume was 20 μL, and the column temperature was 58 °C. Three replicate samples were evaluated in each case.

### Preparation of cell samples for proteins and metabolites extraction

The groups of *Z. mobilis* cells challenged by formic acid, acetic acid, furfural, 5-HMF, phenol, and their combination were named as FA, AA, F, H, P, and C, respectively, and the group of cells grown in the absence of inhibitors was named as CK. Six independent sets of harvests for each group were used for biological replicates. The cells at early-logarithmic growth phase with an OD_600_ value of 0.17 ± 0.02 were collected by centrifugation for 5 min at 4 °C and 6000×*g*, then washed twice with pre-chilled phosphate buffer solution (pH 7.2) and once with pre-chilled deionized water, and finally collected by centrifugation. The collected cell pellets were used for protein and metabolite extraction.

### Proteins and metabolites extraction and their derivatization

For protein extraction, the collected cells were first suspended into a lysis buffer (7 M Urea, 2 M Thiourea, 4% CHAPS, 40 mM Tris–HCl, PH 8.5), followed by ultrasonication on ice for 15 min. Samples supplied with 10 μL PMSF (phenylmethanesulfonyl fluoride, 100 mM) solution were kept at 4 °C for 2 h and then centrifuged for 15 min at 4 °C and 30,000×*g*. The supernatant liquor containing extracted proteins of each sample was precipitated by 10% (v/v) Trichloroacetic acid (TCA)/acetone overnight at − 20 °C. Subsequently, the precipitation collected by centrifugation for 15 min at 4 °C and 30,000×*g* was further washed with pre-chilled acetone, precipitated for 30 min at − 20 °C, and again centrifuged for four times. The final protein precipitation was dissolved, reduced, alkylated, and digested using FASP (Filter-Aided Sample Preparation) method [88], and then labeled with iTRAQ reagent (Applied Biosystems, Foster City, CA, USA) according to the manufacturer’s protocol. The labeled samples were combined for further analysis after incubation for 1 h.

For metabolite extraction and derivatization, the collected cells of each sample were quenched using a pre-cooled 40% ethanol/0.8% sodium chloride solution (− 20 °C) [89]. Each sample was centrifuged for 10 min at − 16 °C and 4000×*g*, and the cell pellets were extracted with 400 uL extraction liquid (3:1 mixture of methanol and chloroform) with an addition of 20 μL l-2-chlorophenylalanine (1 mg/mL stock in dH_2_O) as an internal standard. The mixture was homogenized in a ball mill for 4 min followed by ultrasonication on ice for 5 min. Afterward, the supernatant was separated by centrifugation for 20 min at 4 °C and 12,000×*g* and transferred to a new tube. For QC (quality control) sample,a 40-μL solution was taken from each sample and subsequently added with 10-μL FAMEs (a standard mixture of fatty acid methyl esters, 1 mg/mL C8–C16 and 0.5 mg/mL C18–C24 in chloroform). Then, all the samples were dried in a vacuum concentrator prior to incubation for 30 min at 80 °C with the addition of 30-μL methoxyamination reagent (20 mg/mL in pyridine). Lastly, a 40-μL BSTFA (bis (trimethylsilyl) trifluoroacetamide) regent containing 1% (v/v) TMCS (trimethylchlorosilane) was added to each sample, and the production after incubated for 2 h at 70 °C was used for GC–MS analysis.

### High-pH reverse-phase separation of peptides

The peptide mixture was resolved in a 20-mM ammonium formate buffer A (20-mM ammonium formate in pure water, pH 10.0) and fractionated by high-pH reverse-phase separation using LC-20AB HPLC system (Shimadzu, Japan) with a 4.6 mm × 150 mm, Gemini-NX 5u C18 110A column (Phenomenex, Guangzhou, China). High-pH reverse-phase separation was performed using a linear gradient starting from 5% buffer B to 80% buffer B in 30 min (buffer B: 20 mM ammonium formate in 100% acetonitrile, pH 10.0). Sixteen fractions were collected and dried in a vacuum concentrator rotation vacuum (Christ RVC 2-25, Christ, Germany). Fifty microliter buffer C (0.1% formic acid in 5% acetonitrile) (TEDIA, Fairfield, USA) was added to each dried fraction for RPLC-MS/MS (reversed-phase liquid chromatography-mass spectrum/mass spectrum) analysis.

### RPLC-MS/MS analysis of peptides

The iTRAQ-labeled fractions in buffer C were loaded and separated on a Nano-flow 1D-plus Eksigent HPLC system (Eksigent Technologies, USA) coupled to a Triple TOF 5600 mass spectrometer (AB SCIEX, Foster City, CA, USA). Ten microliter peptide sample was loaded onto the trap column (Thermo Scientific Acclaim PepMap C18, 100 μm × 2 cm), with a flow rate of 10 μL/min for 3 min and subsequently separated on the analytical column (75 μm × 15 cm, Acclaim PepMap C18). For MS analysis, the Triple TOF 5600 system was used in Information Dependent Acquisition Mode. MS spectra were acquired across the range of 350–1250 m/z in high-resolution mode (> 30,000), using 0.25-s accumulation time per spectrum. Twenty most abundant precursor ions per cycle were chosen for fragmentation from each MS spectrum with 0.1-s minimum accumulation time for each precursor. Tandem mass spectra were recorded in high sensitivity mode (resolution > 15,000), with turned on rolling collision and iTRAQ reagent collision energy adjustment. Peptides above a two-count threshold selection in MS/MS analysis were dynamically excluded for 12 s with ± 50 ppm mass tolerance to detect more peptides.

### Protein identification and data analysis

The MS/MS-based raw data files were converted into mascot generic format (MGF) files by ProteinPilot™ Software 4.5 (AB SCIEX, Foster City, CA, USA) and submitted to Mascot_2.5.1 software (Matrix Science, London, UK) to search against the *Zymomonas mobilis* subsp. Mobilis database from the UniProt database (http://www.uniprot.org/uniprot/). Peptide identifications were accepted if they could achieve a false discovery rate (FDR) < 1.0%. At least two peptides with 95% confidence were considered for protein quantification.

The peptide used in iTRAQ quantification was automatically selected by the pro group algorithm to calculate the reported peak area, error factor, and *p* value. To minimize false positive results, a strict cutoff for protein identification was applied with an unused ProtScore ≥ 1.3, which corresponded to a confidence limit of 95%. For data analysis, protein quantification data with a fold change of > 1.5 or < 0.67 and corrected *p* value < 0.05 was selected as the significantly differentially expressed protein (DEP). Bioinformatics analyses of these DEPs were conducted by Kyoto Encyclopedia of Genes and Genomes (KEGG), Gene Ontology (GO), and UniProt analysis. KEGG Pathways with *p* value < 0.05 were recognized as significantly changed ones. Interaction networks of protein to protein and protein to metabolic pathway were constructed by using the String v9.1 [90] and the Cytoscape_v3.5.1 software [91], respectively.

### GC–MS analysis of metabolites

The GC–MS analysis was performed using an Agilent 7890 gas chromatograph system (Agilent Technologies, Palo Alto, CA, USA) coupled with a Pegasus HT TOF mass spectrometer (LECO Corporation, St. Joseph, MI, USA). The system utilized a DB-5MS capillary column (30 μm × 250 μm inner diameter, 0.25 μm film thickness; J&W Scientific, Folsom, CA, USA) coated with 5% diphenyl cross-linked with 95% dimethylpolysiloxane. Helium was used as the carrier gas with a flow rate of 1 mL/min, and the injection volume was 1 μL without a split. The initial temperature was kept at 50 °C for 1 min, then raised to 300 °C at a rate of 10 °C/min, and maintained for 9 min at 300 °C. The injection, transfer line, and ion source temperatures were 280, 270, and 220 °C, respectively. The energy was − 70 eV in electron impact mode. The mass spectrometry data were acquired in a full-scan mode with the m/z range of 50–500 at a rate of 20 spectra per second after a solvent delay of 460 s.

### Metabolites identification and data analysis

Chroma TOF 4.3X software (LECO Corporation, USA) and LECO-Fiehn Rtx5 database were used for raw peaks exacting, data baselines filtering and calibration, peak alignment, deconvolution analysis, peak identification, and peak area integration [92]. The RI (retention time index) method was used in the peak identification according to the RI of FAMEs in QC sample, and the RI tolerance was 5,000. All metabolomic profile data were normalized based on the internal standard compound. Then, data were imported into the SIMCA-P program (version 13.0, Umetrics) for principal component analysis (PCA) and partial least squares discriminate analysis (OPLS-DA). The parameters R2X, R2Y, and Q2Y of the models were analyzed to ensure the quality of the multivariate models. Here, R2 indicates how well the variation of a variable is explained using the predictive components, while Q2 indicates how well a variable can be predicted. Well-modeled variables have R2X, R2Y, and Q2Y values of more than 0.50. Using variable importance to projection (VIP) values, the list of metabolites was iteratively reduced to the absolute minimum required to maintain the strength of model parameters. Metabolite with VIP value more than 1.00 is defined as a differential metabolite. Furthermore, the metabolic pathway analysis of the metabolites was conducted using MetaboAnalyst, a comprehensive tool for metabolomics analysis and interpretation [93].

## Additional files

**Additional file 1: Text S1.** Distribution of gene ontology categories and KEGG pathways of the differential expression proteins and metabolites. **Fig. S1** OD values of the cell cultures under different concentrations of inhibitors. **Fig. S2** Cell growth, residual glucose, and ethanol production of the cell cultures under different concentrations of inhibitors. **Fig. S3** Numbers of differential expression proteins in the groups treated by different inhibitors. **Fig. S4** PCA analysis of the total metabolites identified in the inhibitor-treated groups and control. **Fig. S5** Metabolic pathways and Gene Ontology categories that the differentially expressed proteins were mainly involved in**. Fig. S6** Documented interactions based on String 9.05 database for the differentially expressed proteins shared by all the inhibitor conditions.

**Additional file 2: Sheet S1.** A total of 1366 proteins identified in each inhibitor-treated group and the control.

**Additional file 3: Sheet S2.** A total of 168 metabolites identified in each inhibitor-treated group and the control.

**Additional file 4: Table S1.** Partial biomarker proteins identified in each inhibitor-treated group.

## Abbreviations

ED: Entner–Doudoroff
5-HMF: 5-hydroxymethylfurfural
FA: cells treated by formic acid
AA: cells treated by acetic acid
F: cells treated by furfural
H: cells treated by 5-HMF
P: cells treated by phenol
C: cells treated by combined inhibitors
MIC: minimum inhibitory concentration
CMIC: combination of the MIC
OD600: optical density at wavelength (λ) 600 nm
HPLC: high-performance liquid chromatography
CICC: China Center of Industrial Culture Collection
