# Supplementary material for: Proteomic and metabolomic analysis of the cellular biomarkers related to inhibitors tolerance in Zymomonas mobilis ZM4
Source: Biotechnol Biofuels. 2018 Oct 16;11:283. doi: 10.1186/s13068-018-1287-5 (PMC6190654; doi:10.1186/s13068-018-1287-5)
Supplement: Supplementary file 1 — Additional file 1: Text S1. Distribution of gene ontology categories and KEGG pathways of the differential expression proteins and metabolites. Fig. S1 OD values of the cell cultures under different concentrations of inhibitors. Fig. S2 Cell growth, residual glucose, and ethanol production of the cell cultures under different concentrations of inhibitors. Fig. S3 Numbers of differential expression proteins in the groups treated by different inhibitors. Fig. S4 PCA analysis of the total metabolites identified in the inhibitor-treated groups and control. Fig. S5 Metabolic pathways and Gene Ontology categories that the differentially expressed proteins were mainly involved in. Fig. S6 Documented interactions based on String 9.05 database for the differentially expressed proteins shared by all the inhibitor conditions. [file 13068_2018_1287_MOESM1_ESM.doc]

**Proteomic and metabolomic analysis of the cellular biomarkers related to inhibitors tolerance in *Zymomonas mobilis* ZM4**

Dongdong Chang^1^, Zhisheng Yu^1^*, Zia Ul Islam^1, 2^, W. Todd French^3^, Yiming Zhang^4^, Hongxun Zhang^1^

1. College of Resources and Environment, University of Chinese Academy of Sciences, Beijing 100049, P. R. China

2. Department of Sustainable Bioproducts, Mississippi State University, Mississippi State, MS 39762, USA

3. Dave C. Swalm School of Chemical Engineering, Mississippi State University, P.O. Box 9595, MS 39762, USA

4. Environmental Protection Bureau, Shunyi District, Beijing 100049, P. R. China

All correspondence should be addressed to:

Prof. Zhisheng Yu

College of Resources and Environment

University of Chinese Academy of Sciences,

19 A Yuquan Road, Shijingshan District

Beijing 100049, P. R. China

E-mail: [yuzs@ucas.ac.cn](mailto:yuzs@ucas.ac.cn)

Tel.: +86 10 88256057

Fax: +86 10 88256057;

**This file includes:**

**Text S1** Distribution of gene ontology categories and KEGG pathways of the differential expression proteins and metabolites

**Fig. S1** OD values of the cell cultures under different concentrations of inhibitors

**Fig. S2** Cell growth, residual glucose, and ethanol production of the cell cultures under different concentrations of inhibitors

**Fig. S3** Numbers of differential expression proteins in the groups treated by different inhibitors

**Fig. S4** PCA analysis of the total metabolites identified in the inhibitor-treated groups and control

**Fig. S5** Metabolic pathways and Gene Ontology categories that the differentially expressed proteins were mainly involved in

**Fig. S6** Documented interactions based on String 9.1 database for the differentially expressed proteins shared by all the inhibitor conditions

**Text S1: Distribution of gene ontology categories and KEGG pathways of the differential expression proteins and metabolites**

According to gene ontology (GO) categories, the differential expression proteins (DEPs) of each group were respectively classified by three individual ontologies: biological process, cellular component, and molecular function. Most of the DEPs mainly participated in the metabolic process, constituted the cell part, and played roles in the catalytic activity, corresponding to the above three ontologies. For example, the DEPs involved in the metabolic process, according to the biological process categories, accounted for 54.50 %, 60.87 %, 61.66 %, 57.85 %, 54.25 %, and 62.47 % in groups FA, AA, F, H, P, and C (FA, cells treated by formic acid; AA, cells treated by acetic acid; F, cells treated by furfural; H, cells treated by 5-HMF; P, cells treated by phenol; C, cells treated by combined inhibitors), respectively; the DEPs as certain components of the cell part, according to the cellular component categories, accounted for 16.93 %, 15.22 %, 19.37 %, 18.02 %, 17.22 %, and 16.56 % in these groups, respectively; the DEPs playing roles in the catalytic activity, according to the molecular function categories, accounted for 71.43 %, 77.54 %, 68.77 %, 65.12 %, 63.68 %, and 68.43 % in these groups, respectively.

Also, KEGG pathway analysis showed that the DEPs of these six groups were mostly involved in metabolic pathways, biosynthesis of secondary metabolites pathway, biosynthesis of antibiotics pathway, microbial metabolism in diverse environments pathway, and biosynthesis of amino acids pathway. DEPs involved in these five pathways accounted for 25.93 %, 14.81 %, 12.17 %, 11.11 %, and 8.99 % of the total DEPs in group FA, respectively; 35.51 %, 23.19 %, 18.84 %, 16.67 %, and 15.94 % in group AA, respectively; 26.88 %, 18.07 %, 15.13 %, 10.50 %, and 13.87 % in group F, respectively; 29.07 %, 16.28 %, 13.37 %, 8.14 %, and 9.88 % in group H, respectively; 28.54 %, 14.62 %, 11.32 %, 8.02 %, and 8.25 % in group P, respectively; 32.67 %, 16.78 %, 14.35 %, 11.70 %, and 10.60 % in group C, respectively.

In addition, pathway analysis showed the enriched KEGG pathways of the biomarker metabolites, which showed a similar distribution with that of proteomics results, were mainly metabolic pathways, biosynthesis of secondary metabolites pathway, biosynthesis of antibiotics pathway, microbial metabolism in diverse environments pathway, and ABC transporters pathway. The metabolites involved in above five pathways accounted for respective 84.21 %, 52.63 %, 47.39 %, 42.11 %, and 42.11 % of all the biomarker metabolites in group FA; respective 72.73 %, 50.00 %, 50.00 %, 27.27 %, and 45.45 % in group AA; respective 76.92 %, 53.85 %, 46.15 %, 34.62 %, and 50.00 % in group F; respective 75.00 %, 62.50 %, 62.50 %, 50.00 %, and 56.25 % in group H; respective 82.35 %, 70.59 %, 64.71 %, 52.94 %, and 47.06 % in group P; respective 73.68 %, 63.16 %, 57.89 %, 47.37 %, and 42.11 % in group C.

**
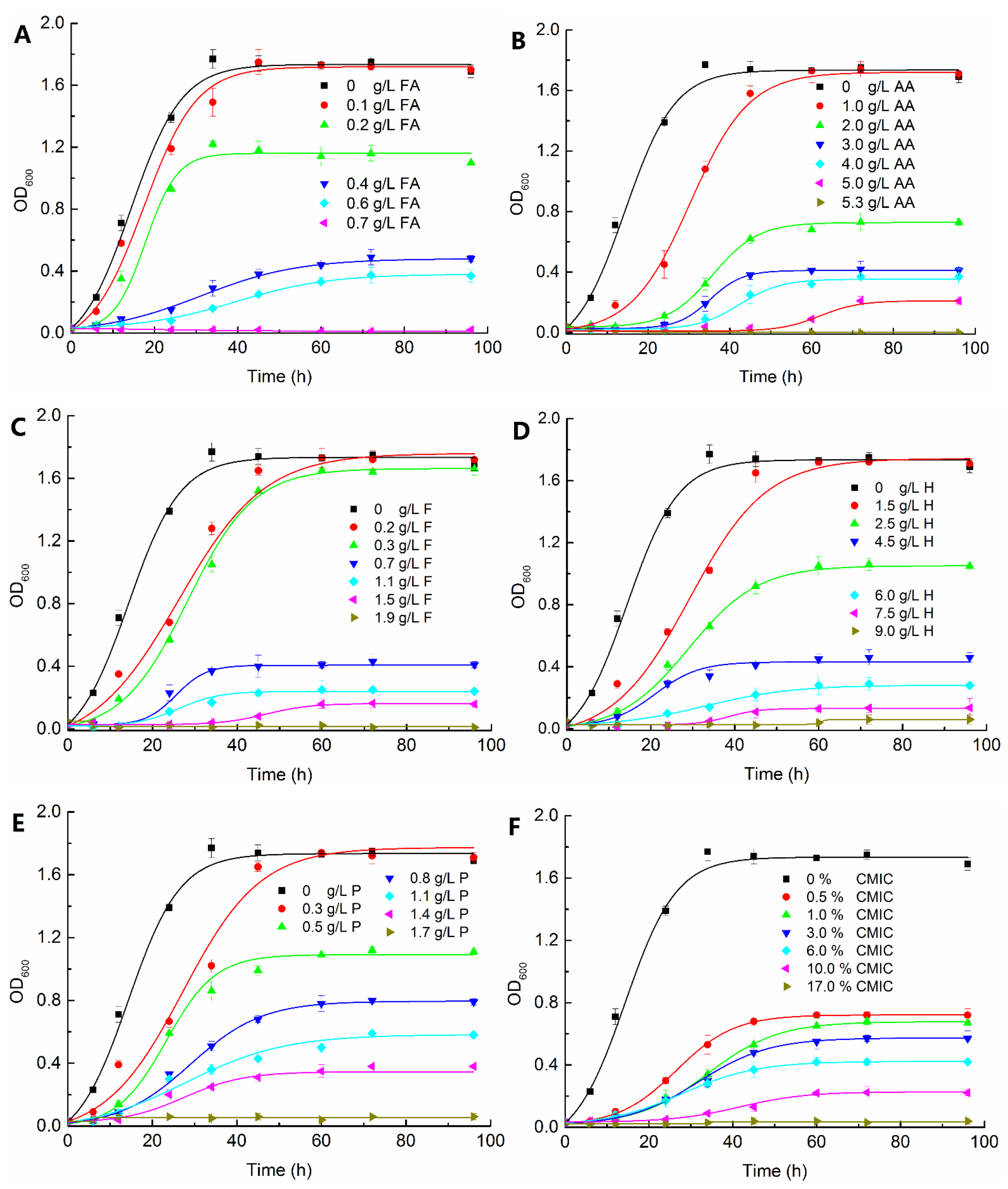
**

**Fig. S1 Cell growth of *Z. mobilis* ZM4 under different concentrations of inhibitors.** **A**) FA, cells treated by formic acid; **B**) AA, cells treated by acetic acid; **C**) F, cells treated by furfural; **D**) H, cells treated by 5-HMF; **E**) P, cells treated by phenol; **F**) CMIC, cells treated by combined inhibitors, and 100 % CMIC denotes the combination of 1X minimal inhibition concentration of each inhibitor.

**
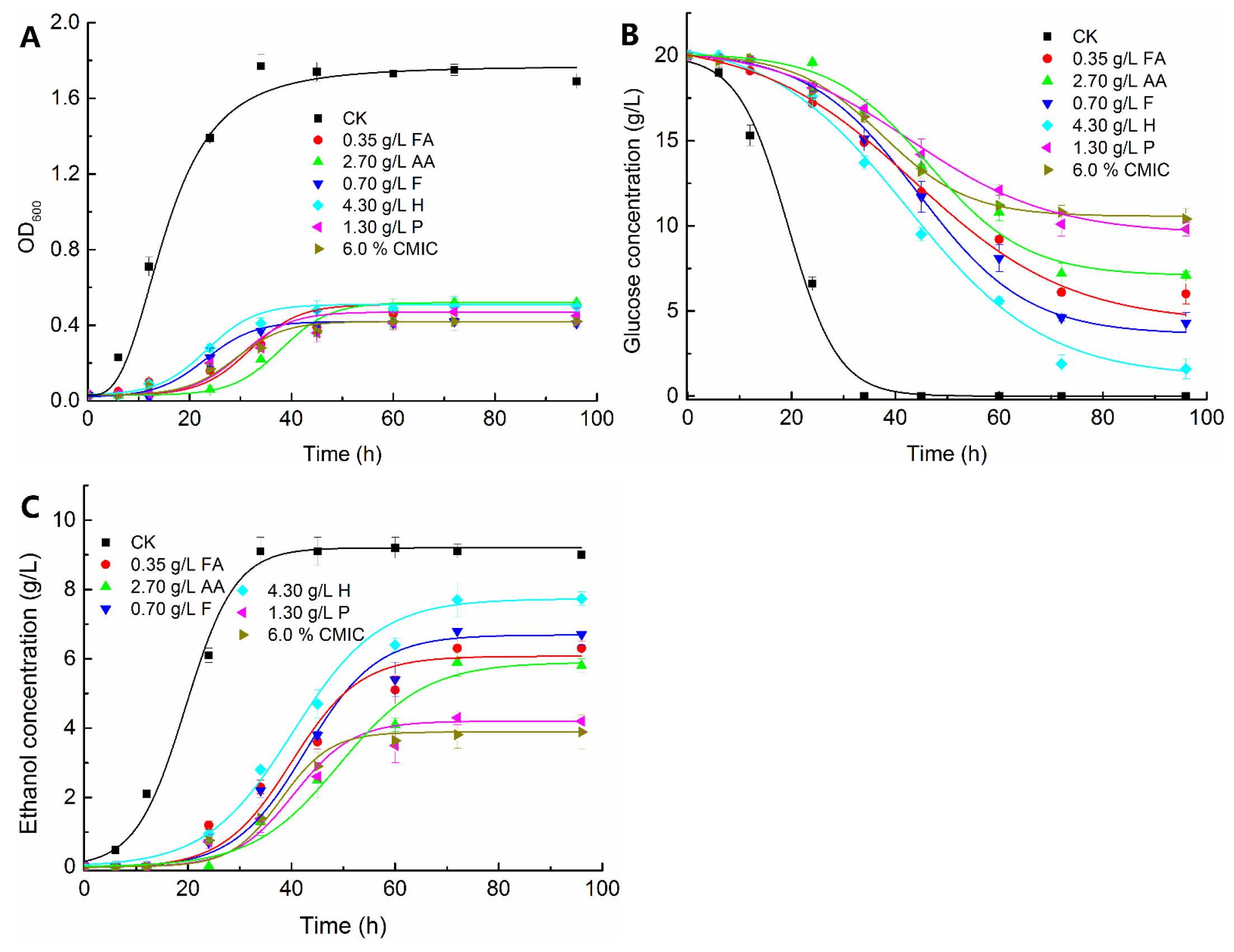
**

**Fig. S2 Cell growth, residual glucose, and ethanol production of the cell cultures under different concentrations of inhibitors.** These concentrations of the inhibitors were chosen for further proteomics and metabolomics analysis. **A** Cell growth of *Z. mobilis* ZM4. **B** Residual glucose in the cultures. **C** Ethanol concentration in the cultures. FA, cells treated by formic acid; AA, cells treated by acetic acid; F, cells treated by furfural; H, cells treated by 5-HMF; P, cells treated by phenol; CMIC, cells treated by combined inhibitors, and 100 % CMIC denotes the combination of 1X minimal inhibition concentration of each inhibitor.

**
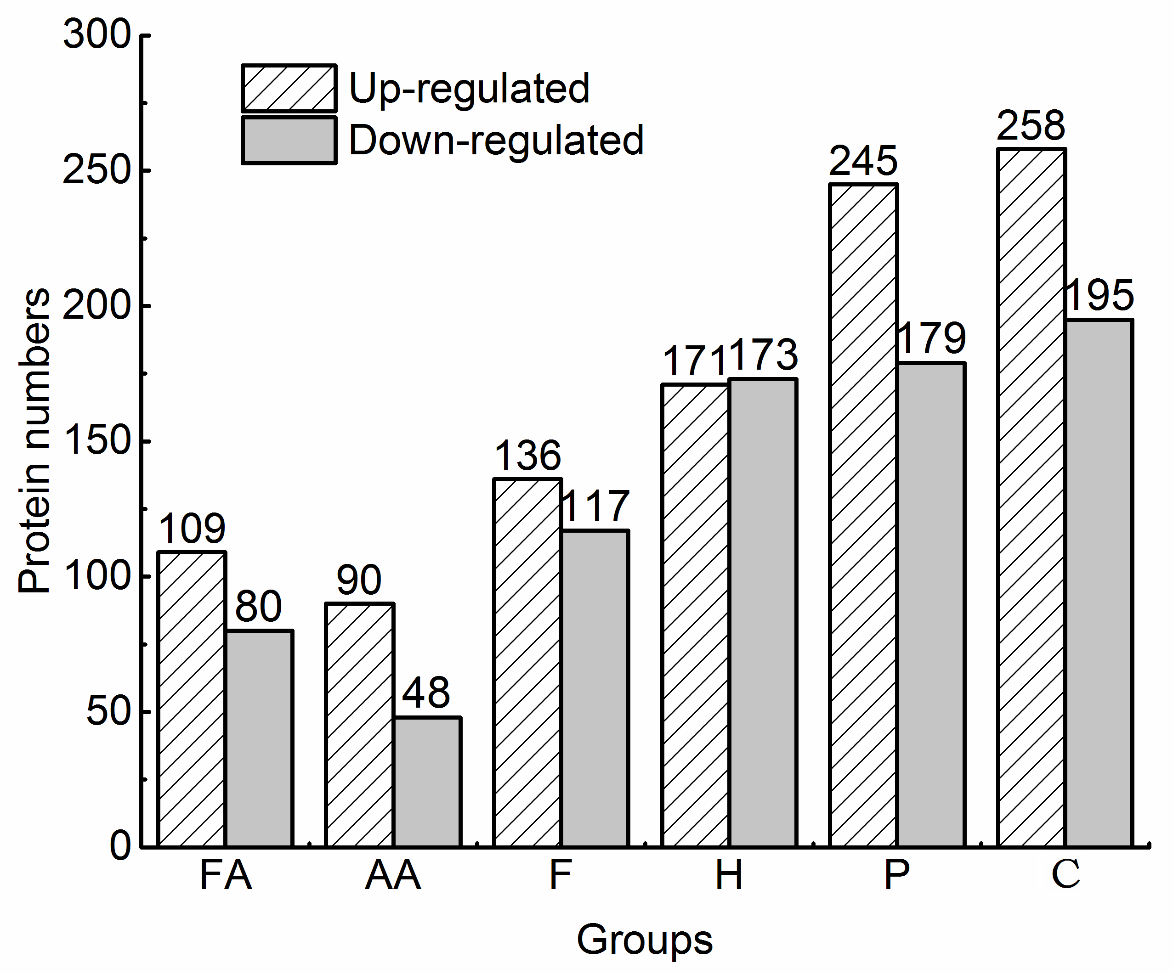
**

**Fig. S3. Numbers of differential expression proteins in the groups treated by different inhibitors.** FA, cells treated by formic acid; AA, cells treated by acetic acid; F, cells treated by furfural; H, cells treated by 5-HMF; P, cells treated by phenol; C, cells treated by combined inhibitors.


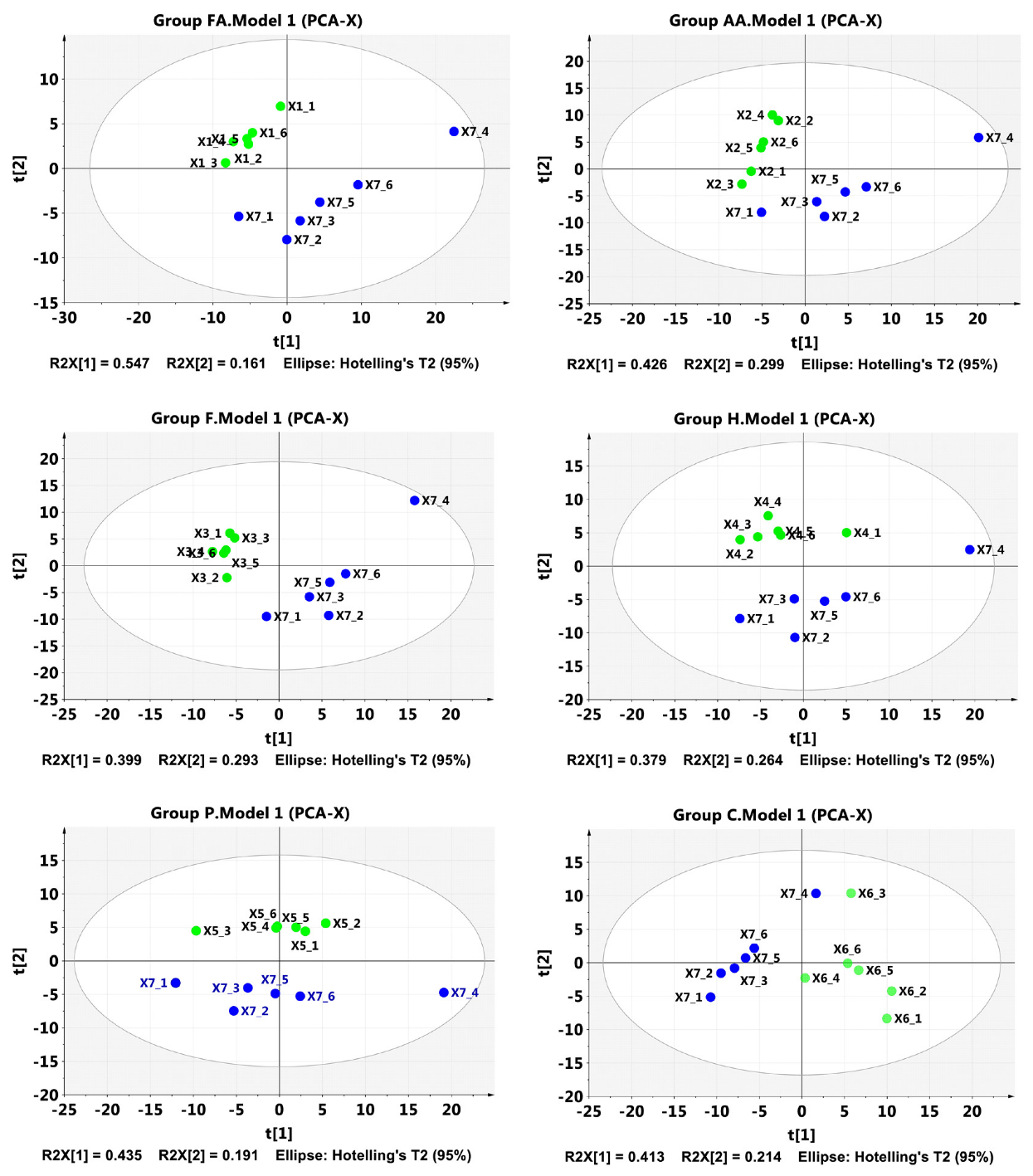


**Fig. S4. PCA analysis of the total metabolites identified in the inhibitor-treated groups and control.** X1_1 to X1_6 were six duplications of metabolites obtained in formic acid treated group, X2_1 to X2_6 were those in acetic acid treated group, X3_1 to X3_6 were those in furfural treated group, X4_1 to X4_6 were those in acetic acid treated group, X5_1 to X5_6 were those in 5-HMF treated group, X6_1 to X6_6 were those in phenol treated group, and X7_1 to X7_6 were those in combined inhibitors treated group.


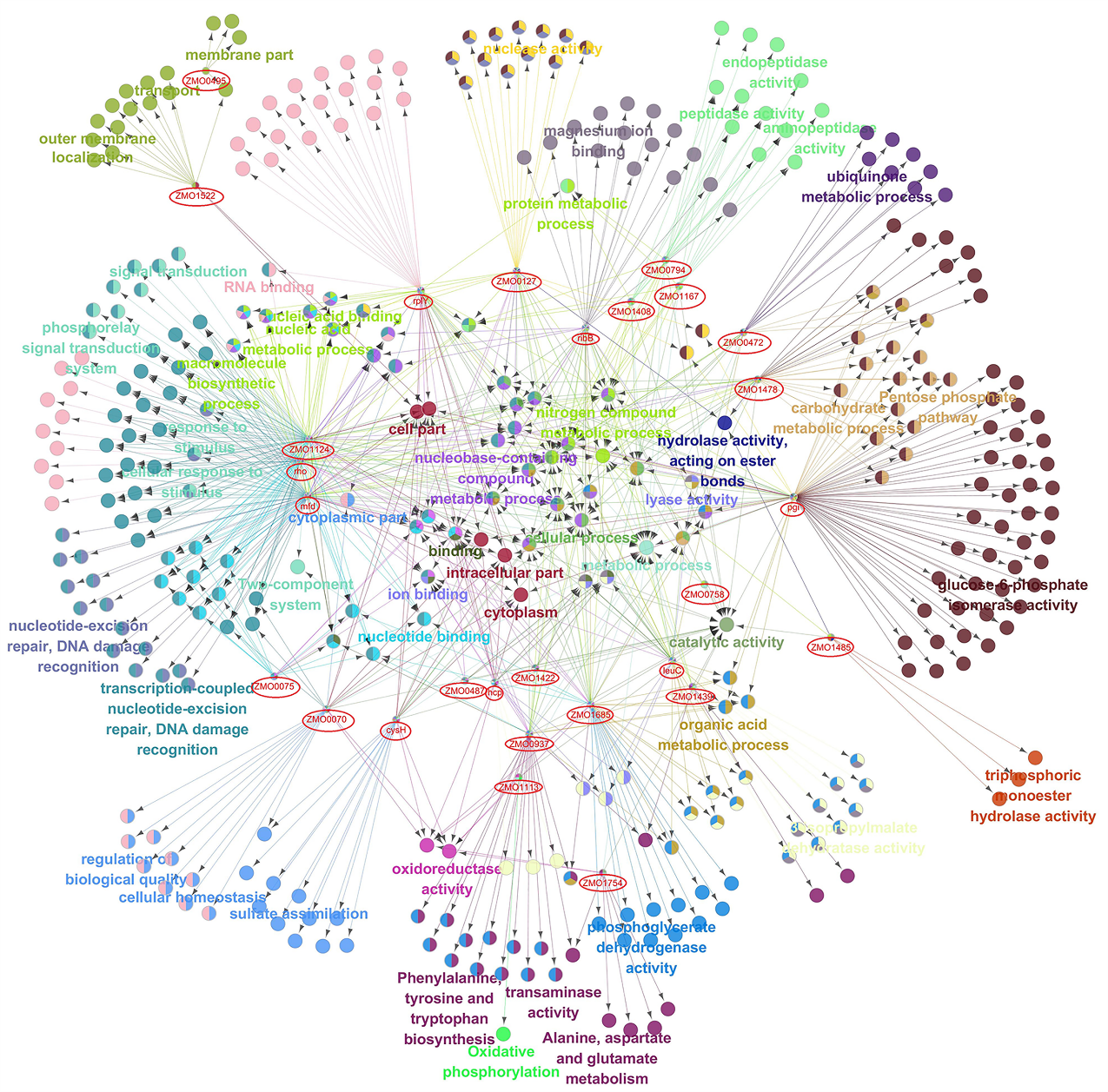


**Fig. S5 Metabolic pathways and Gene Ontology categories that the differentially expressed proteins were mainly involved in.** Biomarker proteins are shown as their gene names labeled with red ellipses.


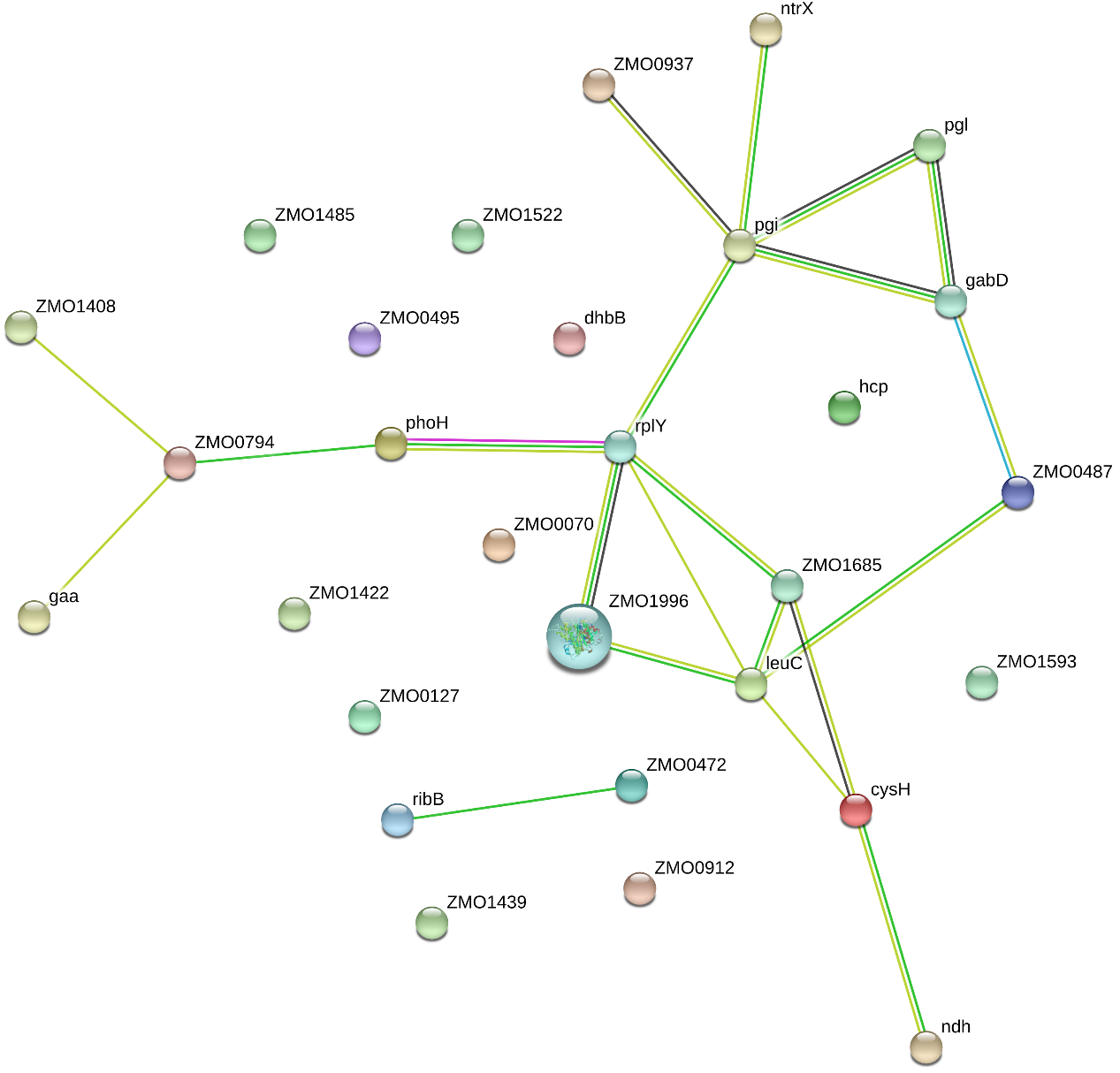


**Fig. S6 Documented interactions based on String 9.1 database for the differentially expressed proteins shared by all the inhibitor conditions.** A greater the number of lines associated with the connection, indicates a greater level of confidence in the association. The network nodes are proteins. The edges represent the predicted functional associations. An edge may be drawn with up to 7 differently colored lines - these lines represent the existence of the seven types of evidence used in predicting the associations. A red line indicates the presence of fusion evidence; a green line - neighborhood evidence; a blue line - coocurrence evidence; a purple line - experimental evidence; a yellow line - textmining evidence; a light blue line - database evidence; a black line - coexpression evidence.
