# Supplementary material for: Proteomic and metabolomic analysis of the cellular biomarkers related to inhibitors tolerance in Zymomonas mobilis ZM4
Source: Biotechnol Biofuels. 2018 Oct 16;11:283. doi: 10.1186/s13068-018-1287-5 (PMC6190654; doi:10.1186/s13068-018-1287-5)
Supplement: Supplementary file 4 — Additional file 4: Table S1. Partial biomarker proteins identified in each inhibitor-treated group. [file 13068_2018_1287_MOESM4_ESM.docx]

**Proteomic and metabolomic analysis of the cellular biomarkers related to inhibitors tolerance in *Zymomonas mobilis* ZM4**

Dongdong Chang^1^, Zhisheng Yu^1^*, Zia Ul Islam^1, 2^, W. Todd French^3^, Yiming Zhang^4^, Hongxun Zhang^1^

1. College of Resources and Environment, University of Chinese Academy of Sciences, Beijing 100049, P. R. China

2. Department of Sustainable Bioproducts, Mississippi State University, Mississippi State, MS 39762, USA

3. Dave C. Swalm School of Chemical Engineering, Mississippi State University, P.O. Box 9595, MS 39762, USA

4. Environmental Protection Bureau, Shunyi District, Beijing 100049, P. R. China

All correspondence should be addressed to:

Prof. Zhisheng Yu

College of Resources and Environment

University of Chinese Academy of Sciences,

19 A Yuquan Road, Shijingshan District

Beijing 100049, P. R. China

E-mail: [yuzs@ucas.ac.cn](mailto:yuzs@ucas.ac.cn)

Tel.: +86 10 88256057

Fax: +86 10 88256057;

**This file includes:**

**Table S1** Partial biomarker proteins identified in each inhibitor-treated group

**Table S1** Partial biomarker proteins identified in each inhibitor-treated group

| Proteins | Locus tag no. | Annotation | Expression pattern in relevant groups | | | | | |
| --- | --- | --- | --- | --- | --- | --- | --- | --- |
|  |  |  | FA | AA | F | H | P | C |
| *Proteins related to cell wall/membrane/envelope biogenesis and cell motility* | | | | | | | | |
| ZMO0082 | ZMO0082 | Chemotaxis protein methyltransferase |  |  |  |  | D |  |
| ZMO0083 | ZMO0083 | CheA signal transduction histidine kinase |  |  |  |  |  | D |
| ZMO0085 | ZMO0085 | Methyl-accepting chemotaxis sensory transducer | D | D |  |  |  | D |
| ZMO0128 | ZMO0128 | TonB-dependent receptor | D | D |  | D | D | D |
| ZMO0166 | ZMO0166 | Peptidoglycan-associated lipoprotein | U |  | U | U | U | U |
| IspG | ZMO0180 | 4-hydroxy-3-methylbut-2-en-1-yl diphosphate synthase |  |  |  |  |  | D |
| ZMO0188 | ZMO0188 | TonB-dependent siderophore receptor |  |  |  |  |  | D |
| ZMO0202 | ZMO0202 | Methyl-accepting chemotaxis sensory transducer |  |  |  |  | D |  |
| ZMO0282 | ZMO0282 | Efflux transporter, RND family, MFP subunit |  |  |  |  | U |  |
| ZMO0283 | ZMO0283 | Efflux pump membrane transporter | D | D |  | U | U |  |
| ZMO0285 | ZMO0285 | RND efflux system, outer membrane lipoprotein |  |  |  |  | U |  |
| ZMO0487 | ZMO0487 | HpcH/HpaI aldolase | D |  |  |  | D | D |
| IspB | ZMO0564 | Polyprenyl synthetase |  |  |  |  |  | D |
| FlgK | ZMO0605 | Flagellar hook-associated protein |  |  |  | D |  |  |
| FlgI | ZMO0607 | Flagellar P-ring protein |  |  |  |  | D | D |
| FlgH | ZMO0608 | Flagellar L-ring protein |  |  |  |  |  | D |
| ZMO0611 | ZMO0611 | Flagellar basal body protein |  |  |  |  |  | D |
| FlhA | ZMO0624 | Flagellar biosynthesis protein |  |  | D | D | D | D |
| FliS | ZMO0652 | Flagellar protein |  |  |  |  |  | D |
| ZMO0778 | ZMO0778 | Acriflavin resistance protein |  |  | D | D | D |  |
| ZMO0779 | ZMO0779 | Efflux transporter, RND family, MFP subunit |  |  | D | D | D |  |
| ZMO0780 | ZMO0780 | RND efflux system, outer membrane lipoprotein |  |  |  | D |  |  |
| FtsA | ZMO0836 | Cell division protein |  |  | U | U |  | U |
| IspA | ZMO0855 | Polyprenyl synthetase |  |  | D | D |  |  |
| Shc | ZMO0872 | Squalene-hopene cyclase | D |  | D | D |  | D |
| HpnH | ZMO0874 | Hopanoid biosynthesis associated radical SAM protein | D |  | D | D | D | D |
| IspH | ZMO0875 | 4-hydroxy-3-methylbut-2-enyl diphosphate reductase |  |  | D | D | D | D |
| CheB | ZMO0878 | Chemotaxis response regulator protein | D |  | D |  | D | D |
| ZMO0908 | ZMO0908 | Lipopolysaccharide biosynthesis protein |  |  | D |  |  | D |
| ZMO0964 | ZMO0964 | RND efflux system, outer membrane lipoprotein |  |  | U |  |  |  |
| ZMO0965 | ZMO0965 | Efflux pump membrane protein |  |  | U |  |  |  |
| HpnJ | ZMO0973 | Hopanoid biosynthesis associated radical SAM protein | D | D |  | D |  | D |
| ZMO0979 | ZMO0979 | TonB-dependent receptor |  | D | D | D | D |  |
| ZMO1017 | ZMO1017 | ABC transporter related protein |  |  |  |  | D |  |
| ZMO1029 | ZMO1029 | ABC transporter related protein |  |  |  |  | U | D |
| ZMO1083 | ZMO1083 | Cellulose synthase catalytic subunit |  |  |  |  | U |  |
| IspDF | ZMO1128 | Bifunctional enzyme IspD/IspF |  | U | U | U | U | U |
| Dxr | ZMO1150 | 1-deoxyxylulose-5-phosphate reductoisomerase |  |  |  | D |  |  |
| ZMO1207 | ZMO1207 | Apolipoprotein N-acyltransferase |  |  |  |  |  | U |
| Dxs1 | ZMO1234 | 1-deoxyxylulose-5-phosphate synthase 1 | D | D | D | D |  | D |
| ZMO1355 | ZMO1355 | ABC transporter related protein |  |  | D | D |  | D |
| ZMO1430 | ZMO1430 | Efflux transporter, RND family, MFP subunit |  |  |  |  | U |  |
| ZMO1439 | ZMO1439 | Apolipoprotein N-acyltransferase | D | D | D | D | D | D |
| ZMO1522 | ZMO1522 | TonB-dependent receptor | D | D | D | D | D | D |
| ZMO1529 | ZMO1529 | Efflux transporter, RND family, MFP subunit |  |  |  |  | U |  |
| ZMO1590 | ZMO1590 | ABC transporter related protein |  |  |  |  | U | U |
| ZMO1694 | ZMO1694 | TonB-dependent receptor | D |  |  | D | D | D |
| ZMO1701 | ZMO1701 | Outer-membrane lipoprotein carrier protein |  |  |  |  | U |  |
| ZMO1717 | ZMO1717 | TonB family protein |  |  | D | D | D |  |
| *Proteins related to energy production and conversion* | | | | | | | | |
| ZMO0191 | ZMO0191 | DSBA oxidoreductase |  | U |  |  | U | U |
| AtpH | ZMO0238 | F-type ATPase subunit delta | U | U |  |  | U | U |
| AtpA | ZMO0239 | F-type ATPase subunit alpha | U | U |  | U | U | U |
| AtpG | ZMO0240 | F-type ATPase subunit gamma | U | U |  |  |  | U |
| AtpD | ZMO0241 | F-type ATPase subunit beta |  |  |  |  |  | U |
| ZMO0242 | ZMO0242 | H^+^ transporting two-sector ATPase delta/epsilon subunit |  |  |  |  |  | U |
| ZMO0441 | ZMO0441 | ATP12 ATPase | U | U |  |  | U | U |
| ZMO0915 | ZMO0915 | Copper-translocating P-type ATPase |  | U | U | U |  | U |
| Ndh | ZMO1113 | Pyridine nucleotide-disulfide oxidoreductase/ type II NADH dehydrogenase | U | U | U | U | D | U |
| ZMO1184 | ZMO1184 | Electron-transferring-flavoprotein dehydrogenase | D |  |  | D |  | D |
| ZMO1419 | ZMO1419 | 4-hydroxybenzoate octaprenyltransferase |  |  |  |  | U | U |
| ZMO1480 | ZMO1480 | Electron transfer flavoprotein alpha/beta-subunit |  |  |  |  | U | U |
| Ppa | ZMO1507 | Inorganic pyrophosphatase |  |  | D | D | D | D |
| ZMO1753 | ZMO1753 | Oxidoreductase FAD/NAD(P)-binding domain protein |  |  |  | D | D | D |
| ZMO1754 | ZMO1754 | Aldehyde dehydrogenase | U | U | U | U | D | U |
| ZMO1842 | ZMO1842 | Flavin transferase |  |  |  |  | U | U |
| ZMO1844 | ZMO1844 | Oxidoreductase domain protein | U |  |  | U |  |  |
| GshB | ZMO1913 | Glutathione synthetase |  |  |  | U | U | U |
| *Proteins related to DNA replication, recombination, repair, transcription, and RNA translation* | | | | | | | | |
| HrcA | ZMO0015 | Heat-inducible transcription repressor |  | U |  |  | U | U |
| Hpf | ZMO0038 | Ribosome hibernation promoting factor | U |  | U | U |  | U |
| ZMO0127 | ZMO0127 | S1/P1 nuclease | D | D | D | D | D | D |
| ZMO0219 | ZMO0219 | Helicase domain protein | D | D | D | D |  |  |
| PolA | ZMO0227 | DNA polymerase I | D |  | D | D |  | D |
| ZMO0257 | ZMO0257 | Two component transcriptional regulator |  |  |  |  | D | D |
| RpoN | ZMO0274 | RNA polymerase sigma-54 factor |  |  |  |  | U | U |
| ZMO0301 | ZMO0301 | PepSY-associated TM helix domain protein |  |  |  |  | D | D |
| Efp | ZMO0328 | Elongation factor P |  |  | D |  | D | D |
| HflX | ZMO0348 | GTP-binding protein, GTPase |  |  | U |  | U |  |
| UvrB | ZMO0362 | Excinuclease ABC subunit B |  |  | U | U | U | U |
| ParE | ZMO0411 | DNA topoisomerase IV subunit |  |  | D | D |  | D |
| ZMO0430 | ZMO0430 | Pyrimidine 5'-nucleotidase | D |  | D | D |  | D |
| ZMO0472 | ZMO0472 | RpsU-divergently transcribed protein | U | U | U | U | U | U |
| ZMO0478 | ZMO0478 | Two component transcriptional regulator |  |  |  |  | U | U |
| RpsG | ZMO0514 | 30S ribosomal protein S7 |  |  | D | D | D | D |
| RplB | ZMO0520 | 50S ribosomal protein L2 |  |  | D | D |  | D |
| RplV | ZMO0521 | 50S ribosomal protein L22 |  |  | D | D | D | D |
| RpsC | ZMO0522 | 30S ribosomal protein S3 |  |  | D | D |  | D |
| RpsQ | ZMO0525 | 30S ribosomal protein S17 |  |  |  |  | D | D |
| RplN | ZMO0526 | 50S ribosomal protein L14 | D |  | D | D |  | D |
| RplE | ZMO0528 | 50S ribosomal protein L5 | D |  | D | D | D | D |
| RpsM | ZMO0539 | 30S ribosomal protein S13 |  |  | D | D |  | D |
| InfB | ZMO0554 | Translation initiation factor IF-2 |  | D | D | D |  |  |
| NusA | ZMO0556 | Transcription termination protein |  |  | D | D |  | D |
| RimP | ZMO0557 | Ribosome maturation factor |  |  |  | D | D | D |
| RadA | ZMO0589 | DNA repair protein |  |  |  | U | U |  |
| FliA | ZMO0626 | RNA polymerase, sigma 28 subunit |  |  |  | U | U |  |
| AspS | ZMO0715 | Aspartyl-tRNA synthetase |  |  | D | D |  | D |
| NusG | ZMO0724 | LysR family transcriptional regulator |  |  |  | D |  | D |
| RplK | ZMO0725 | 50S ribosomal protein L11 |  |  |  |  | D | D |
| RplA | ZMO0726 | 50S ribosomal protein L1 |  |  | D | D |  | D |
| RplJ | ZMO0727 | 50S ribosomal protein L10 |  |  |  | D | D | D |
| RpoB | ZMO0731 | DNA-directed RNA polymerase subunit beta |  |  | D |  |  |  |
| RpoC | ZMO0732 | DNA-directed RNA polymerase subunit beta' |  |  | D |  |  |  |
| ZMO0734 | ZMO0734 | 3'(2'), 5'-bisphosphate nucleotidase |  |  | U | U |  | U |
| RpoH | ZMO0749 | RNA polymerase sigma factor |  |  | U | U | U | U |
| ZMO0781 | ZMO0781 | LysR family transcriptional regulator |  |  |  | D |  | D |
| GatB | ZMO0782 | Aspartyl/glutamyl-tRNA amidotransferase subunit B | D |  |  |  | D |  |
| RimO | ZMO0807 | Ribosomal protein S12 methylthiotransferase | D |  | D | D | D | D |
| ArgS | ZMO0843 | Arginyl-tRNA synthetase | D | D | D | D |  | D |
| AlaS | ZMO0845 | Alanyl-tRNA synthetase | D |  |  | D |  | D |
| ZMO0850 | ZMO0850 | Sigma54 specific transcriptional regulator, Fis family |  |  | U | U | U | U |
| RpsI | ZMO0883 | 30S ribosomal protein S9 |  |  |  | D | D | D |
| RplM | ZMO0884 | 50S ribosomal protein L13 |  | D | D | D | D | D |
| ZMO0963 | ZMO0963 | TetR family transcriptional regulator | U |  |  | U | U | U |
| ZMO0980 | ZMO0980 | DNA polymerase III |  |  |  | U |  | U |
| RlmN | ZMO1032 | Dual-specificity RNA methyltransferase | D |  | D | D |  | D |
| MetG | ZMO1092 | Methionyl-tRNA synthetase | D |  |  |  |  | D |
| Rnr | ZMO1096 | Ribonuclease R | U |  | U | U | U | U |
| ZMO1124 | ZMO1124 | Fis family transcriptional regulator | U | U | U | U | U | U |
| ZMO1135 | ZMO1135 | Peptidase U62 modulator of DNA gyrase | D | D |  |  |  |  |
| RpmE | ZMO1145 | 50S ribosomal protein L31 |  |  | D |  | D | D |
| Tsf | ZMO1155 | Elongation factor Ts |  |  | D | D |  | D |
| RecA | ZMO1166 | Recombinase A | D |  | D |  | U | D |
| MutM | ZMO1187 | Fapy-DNA glycosylase |  |  |  |  | U | U |
| RplI | ZMO1227 | 50S ribosomal protein L9 |  |  | D | D | D | D |
| RpsD | ZMO1366 | 30S ribosomal protein S4 |  |  | D | D | D | D |
| Era | ZMO1384 | GTPase |  | D | D |  | D | D |
| ZMO1417 | ZMO1417 | DEAD/DEAH box helicase domain protein |  | D | D | D | D | D |
| LeuS | ZMO1435 | Leucyl-tRNA synthetase | U | U | U | U |  |  |
| UvrA | ZMO1588 | Excinuclease ABC subunit A | U |  | U | U | U | U |
| RpoD | ZMO1623 | RNA polymerase sigma factor (Sigma-70) |  |  | D |  | D | D |
| Mfd | ZMO1646 | Transcription-repair-coupling factor | U | U | U | U | U | U |
| ZMO1738 | ZMO1738 | LytTR family transcriptional regulator |  | U |  |  | U | U |
| ZMO1748 | ZMO1748 | ArsR family transcriptional regulator | D |  |  | D |  |  |
| RpsA | ZMO1798 | 30S ribosomal protein S1 | D |  | D | D |  | D |
| MutS | ZMO1907 | DNA mismatch repair protein |  |  |  |  | U | U |
| RplY | ZMO1910 | 50S ribosomal protein L25 (General stress protein CTC) | D | D | D | D | D | D |
| PriA | ZMO1921 | Primosomal protein N' |  |  |  |  | U | U |
| Rho | ZMO1996 | Transcription termination factor | D | D | D | D | D | D |
| RpsS | ZMO2004 | 30S ribosomal protein S19 |  |  | D | D | D | D |
| *Proteins related to posttranslational modification, protein turnover, and chaperones* | | | | | | | | |
| GrpE | ZMO0016 | Heat shock 70 kDa protein cofactor |  |  |  | U | U | U |
| ZMO0070 | ZMO0070 | Glutaredoxin, GrxB family | U | U | U | U | U | U |
| ZMO0145 | ZMO0145 | Peptidase M28 | D | D | \ | \ | D | D |
| HslU | ZMO0247 | ATP-dependent protease ATPase subunit | U | \ | U | U | U | U |
| ZMO0425 | ZMO0425 | FeS assembly ATPase SufC | \ | \ | U | U | U | U |
| ZMO0426 | ZMO0426 | SufBD protein | \ | \ | U | U | U | U |
| DnaK | ZMO0660 | Heat shock 70 kDa protein, chaperone protein | U | U |  | U | U | U |
| DnaJ | ZMO0661 | Chaperone protein |  |  |  |  | U | U |
| ZMO0753 | ZMO0753 | Glutaredoxin 3 | U | \ | U | U | \ | U |
| GlnD | ZMO0766 | Bifunctional uridylyltransferase/uridylyl-removing enzyme | U | \ | \ | \ | U | U |
| ZMO0935 | ZMO0935 | Glutathione S-transferase domain protein | U | U | U | U | \ | U |
| Tig | ZMO0946 | Trigger factor | D | \ | \ | D | D | D |
| ZMO0989 | ZMO0989 | Heat shock protein Hsp20 | \ | \ | \ | U | U | U |
| ZMO1019 | ZMO1019 | Deoxyhypusine synthase-like protein | D | D | \ | D | D | D |
| ZMO1034 | ZMO1034 | Calcium-binding EF-hand-containing protein | U | \ | U | U | U | U |
| ZMO1422 | ZMO1422 | Peptidase M16 domain protein | D | D | D | D | D | D |
| ClpB | ZMO1424 | Chaperone protein | U | U |  | U | U | U |
| ZMO1593 | ZMO1593 | Peptidase M61 domain protein | D | D | D | D | D | D |
| ZMO1690 | ZMO1690 | Chaperone DnaJ domain protein |  |  |  |  | U | U |
| ZMO1705 | ZMO1705 | Thioredoxin domain protein | U | \ | U | U | U | U |
| Prx | ZMO1732 | Alkyl hydroperoxide reductase/ Thiol specific antioxidant | U | \ | \ | U | U | U |
| ZMO1877 | ZMO1877 | Glutamine cyclotransferase | U | U | U | U | \ | U |
| GroES | ZMO1928 | 10 kDa chaperone protein |  |  |  | U | U | U |
| GroEL | ZMO1929 | 60 kDa chaperone protein |  |  |  |  | U |  |
| *Proteins related to biosynthesis of amino acids* | | | | | | | | |
| LeuC | ZMO0105 | Isopropylmalate isomerase | U | U | U | U | U | U |
| TrpD | ZMO0200 | Anthranilate phosphoribosyltransferase | U |  | U | U |  | U |
| TyrC | ZMO0420 | Cyclohexadienyl dehydrogenase |  | D | D |  |  |  |
| ZMO0490 | ZMO0490 | Oligopeptidase B |  |  |  |  | U | U |
| ZMO0493 | ZMO0493 | Glutamine synthetase |  | U | U |  | U | U |
| ZMO0543 | ZMO0543 | Aconitate hydratase |  |  |  |  | D | D |
| TrpF | ZMO0586 | N-(5'-phosphoribosyl) anthranilate isomerase | U |  |  |  | U | U |
| MetZ | ZMO0676 | O-succinylhomoserine sulfhydrylas |  |  | U |  | U | U |
| LeuB | ZMO0677 | 3-isopropylmalate dehydrogenase |  | U | U | U | U | U |
| ZMO0794 | ZMO0794 | Prolyl oligopeptidase |  |  | D | D |  | D |
| ZMO0913 | ZMO0913 | Branched-chain amino acid aminotransferase |  |  |  |  |  | U |
| ZMO0937 | ZMO0937 | Aromatic amino acid transaminase | D | D | D | D | D | D |
| MetE | ZMO1000 | Methionine synthase | U | U |  |  | U |  |
| DapF | ZMO1072 | Diaminopimelate epimAerase | U |  | U | U | U | U |
| ZMO1139 | ZMO1139 | Acetolactate synthase |  | U |  | U | U | U |
| ZMO1140 | ZMO1140 | Acetolactate synthase, small subunit |  | U | U | U | U | U |
| IlvC | ZMO1141 | Ketol-acid reductoisomerase |  | U | U |  |  | U |
| ZMO1167 | ZMO1167 | Peptidase S15 | D | D | D | D | D | D |
| AstB | ZMO1172 | N-succinylarginine dihydrolase |  |  | U | U | U |  |
| ZMO1275 | ZMO1275 | Threonine dehydratase |  |  |  | U | U | U |
| PepA | ZMO1309 | Leucine aminopeptidase |  |  | U | U |  | U |
| ZMO1408 | ZMO1408 | Peptidase S9B dipeptidylpeptidase IV domain protein | D | D | D | D | D | D |
| ZMO1570 | ZMO1570 | Formate acetyltransferase |  |  | D | D |  | D |
| ThrB | ZMO1600 | Homoserine kinase |  |  | U | U |  | U |
| ZMO165 | ZMO1653 | Aspartokinase |  |  |  | D | D | D |
| ZMO1684 | ZMO1684 | Phosphoserine aminotransferase |  |  | U | U |  |  |
| GlyA | ZMO1201 | Serine hydroxymethyltransferase |  | U | U |  |  | U |
| ZMO1347 | ZMO1347 | Threonine aldolase/ amino acid beta-eliminating lyase |  |  |  | U |  |  |
| Asd | ZMO1407 | Aspartate-semialdehyde dehydrogenase |  | U |  |  |  | U |
| HisA | ZMO1501 | Phosphoribosylformimino-5-aminoimidazole carboxamide ribotide isomerase |  | U | U |  |  | U |
| ZMO1682 | ZMO1682 | Aminotransferase | U |  |  | U | U | U |
| IlvD | ZMO1792 | Dihydroxy-acid dehydratase | U | U | U |  | U | U |
| ZMO1891 | ZMO1891 | Threonine synthase | U | U |  |  | U | U |
| ZMO1989 | ZMO1989 | Methylated-DNA/protein-cysteine methyltransferase |  |  |  |  | U | U |
| *Proteins related to central carbon metabolism* | | | | | | | | |
| GAP | ZMO0177 | Glyceraldehyde 3-phosphate dehydrogenase |  |  |  |  |  | U |
| PGK | ZMO0178 | Phosphoglycerate kinase | U | U |  |  |  | U |
| FBP | ZMO0179 | Fructose-bisphosphate aldolase | U |  | U | U | U |  |
| ZWF | ZMO0367 | Glucose 6-phosphate dehydrogenase |  | U | D |  | U | U |
| EDD | ZMO0368 | 6-phosphogluconate dehydratase | U | U |  | D | U | U |
| GLK | ZMO0369 | Glucokinase |  |  |  |  |  | U |
| KDHC | ZMO0512 | E3 subunit of α-ketoglutarate dehydrogenase complex |  |  |  |  |  | U |
| AH | ZMO0543 | Aconitate hydratase |  |  |  |  | D | D |
| ICDH | ZMO0544 | Isocitrate dehydrogenase | U | U | U | U |  | U |
| EDA | ZMO0997 | 2-keto-3-deoxy-phosphogluconate aldolase |  |  |  |  |  | U |
| PGI | ZMO1212 | Glucose-6-phosphate isomerase | U | U | U | U | U | U |
| ADHA | ZMO1236 | Alcohol dehydrogenase I | U | U | U | U |  | U |
| PGM | ZMO1240 | Phosphoglyceromutase | U | U |  |  | D | U |
| FUM | ZMO1307 | Fumarase |  |  |  | D |  |  |
| PGL | ZMO1478 | Phosphogluconolactonase | U | U | U | U | D | U |
| PPC | ZMO1496 | Phosphoenolpyruvate carboxylase |  |  |  | D | U | U |
| ENO | ZMO1608 | Enolase |  |  |  |  |  | U |
| ME | ZMO1955 | Malic enzyme | U | U | U |  |  | U |
| CS | ZMO1963 | Citrate synthase | U | U | U |  | U | U |

**Notes:** **FA**, cells treated by formic acid; **AA**, cells treated by acetic acid; **F**, cells treated by furfural; **H**, cells treated by 5-HMF; **P**, cells treated by phenol; **C**: cells treated by combined inhibitors. U, up-regulated; D, down-regulated; “\”, no differential expression or p-value＞0.05.
